# Human embryonic stem cell-derived test systems for developmental neurotoxicity: a transcriptomics approach

**DOI:** 10.1007/s00204-012-0967-3

**Published:** 2012-11-21

**Authors:** Anne K. Krug, Raivo Kolde, John A. Gaspar, Eugen Rempel, Nina V. Balmer, Kesavan Meganathan, Kinga Vojnits, Mathurin Baquié, Tanja Waldmann, Roberto Ensenat-Waser, Smita Jagtap, Richard M. Evans, Stephanie Julien, Hedi Peterson, Dimitra Zagoura, Suzanne Kadereit, Daniel Gerhard, Isaia Sotiriadou, Michael Heke, Karthick Natarajan, Margit Henry, Johannes Winkler, Rosemarie Marchan, Luc Stoppini, Sieto Bosgra, Joost Westerhout, Miriam Verwei, Jaak Vilo, Andreas Kortenkamp, Jürgen Hescheler, Ludwig Hothorn, Susanne Bremer, Christoph van Thriel, Karl-Heinz Krause, Jan G. Hengstler, Jörg Rahnenführer, Marcel Leist, Agapios Sachinidis

**Affiliations:** 1 Department of Biology, University of Konstanz (UKN), 78457 Constance, Germany; 2 OÜ Quretec (Qure), Limited Liability Company, 51003 Tartu, Estonia; 3 Institute of Computer Science, University of Tartu, 50409 Tartu, Estonia; 4 Center of Physiology and Pathophysiology, Institute of Neurophysiology, University of Cologne (UKK), Robert-Koch-Str. 39, 50931 Cologne, Germany; 5 Leibniz Research Centre for Working Environment and Human Factors (IfADo), Technical University of Dortmund, 44139 Dortmund, Germany; 6 Commission of the European Communities (JRC) Joint Research Centre, 1049 Brussels, Belgium; 7 Department of Pathology and Immunology, Geneva Medical Faculty, University of Geneva (UNIGE), 1211 Geneva 4, Switzerland; 8 Brunel University (Brunel), Uxbridge, UB8 3PH UK; 9 Nederlandse Organisatie voor Toegepast Natuurwetenschappelijk Onderzoek (TNO), 2628 VK Delft, The Netherlands; 10 Gottfried Wilhelm Leibniz University (LUH), Institute for Biostatistics, 30167 Hannover, Germany; 11 Department of Statistics, TU Dortmund University , 44221 Dortmund, Germany

**Keywords:** Methylmercury, Valproic acid, Transcription factor, Reproductive toxicity, Alternative testing strategies

## Abstract

Developmental neurotoxicity (DNT) and many forms of reproductive toxicity (RT) often manifest themselves in functional deficits that are not necessarily based on cell death, but rather on minor changes relating to cell differentiation or communication. The fields of DNT/RT would greatly benefit from in vitro tests that allow the identification of toxicant-induced changes of the cellular proteostasis, or of its underlying transcriptome network. Therefore, the ‘human embryonic stem cell (hESC)-derived novel alternative test systems (ESNATS)’ European commission research project established RT tests based on defined differentiation protocols of hESC and their progeny. Valproic acid (VPA) and methylmercury (MeHg) were used as positive control compounds to address the following fundamental questions: (1) Does transcriptome analysis allow discrimination of the two compounds? (2) How does analysis of enriched transcription factor binding sites (TFBS) and of individual probe sets (PS) distinguish between test systems? (3) Can batch effects be controlled? (4) How many DNA microarrays are needed? (5) Is the highest non-cytotoxic concentration optimal and relevant for the study of transcriptome changes? VPA triggered vast transcriptional changes, whereas MeHg altered fewer transcripts. To attenuate batch effects, analysis has been focused on the 500 PS with highest variability. The test systems differed significantly in their responses (<20 % overlap). Moreover, within one test system, little overlap between the PS changed by the two compounds has been observed. However, using TFBS enrichment, a relatively large ‘common response’ to VPA and MeHg could be distinguished from ‘compound-specific’ responses. In conclusion, the ESNATS assay battery allows classification of human DNT/RT toxicants on the basis of their transcriptome profiles.

**Electronic supplementary material:**

The online version of this article (doi:10.1007/s00204-012-0967-3) contains supplementary material, which is available to authorized users.

## Introduction

Reproductive toxicity (RT) testing is one of the technically most challenging fields of toxicology, and there is a huge demand for more cost-effective, faster, and more accurate assays. RT may be caused by chemicals, drugs, pesticides and other compounds that interfere with biological processes essential for reproduction, and it is therefore of large societal concern. It has been estimated that up to 50 % of the animals used for testing in the context of REACH will be required to evaluate RT (Seiler et al. 2011). Currently, this type of safety assessment comprises evaluation of chemical effects on spermatogenesis, oogenesis or the fertilization process. Another large subfield deals with the disturbances of embryo–foetal development and is generally called developmental toxicity (DT) testing.

In the area of RT testing, evaluation of a single compound requires hundreds of animals. If testing of nervous system development and long-term effects are included, even thousands of rats/rabbits are required. Animal testing, for example, following OECD test guidelines 414 (2-generation reproduction), 426 (developmental neurotoxicity (DNT)) or others, often only gives indirect indications of toxicity such as changed numbers of embryo–foetal death, altered foetal weight or the development of anatomical or behavioural abnormalities. To significantly reduce the use of animals and to get further mechanistic insights, in vitro systems modelling critical parts of the foetal development are being explored as alternatives (Adler et al. 2011; Basketter et al. 2012); for instance, the development of initial germ layers from pluripotent cells, and the specification of organ systems such as the central nervous system (CNS) are such critical parts of the development.

The CNS is considered to be one of the most frequent targets of systemic toxicity, with the developing nervous system being particularly susceptible (Klaassen 2010; van Thriel et al. 2012). This susceptibility to DNT is due to a finely orchestrated sequence of complex biological processes, such as proliferation, migration, apoptosis, differentiation, patterning, neurite outgrowth, synaptogenesis, myelination and neurotransmitter synthesis, which are all targets of numerous toxic chemicals (Kadereit et al. 2012). Despite its high relevance, DNT is one of the least studied forms of toxicity (Kadereit et al. 2012; Makris et al. 2009). It is also particularly difficult to study, because DNT is not necessarily caused by cell death. In fact, chemically induced changes in the proportions of neural cells, positioning or connectivity may be sufficient to cause DNT (Kadereit et al. 2012; Kuegler et al. 2010). Currently, DNT is tested according to OECD TG 426, which requires animals to be exposed during gestation and lactation, and the resulting offspring to be analysed for gross neurologic and behavioural abnormalities. However, this complex in vivo test system is too laborious and expensive to allow all the testing needed to provide hazard information for thousands of untested chemicals.

**Fig. 1:**
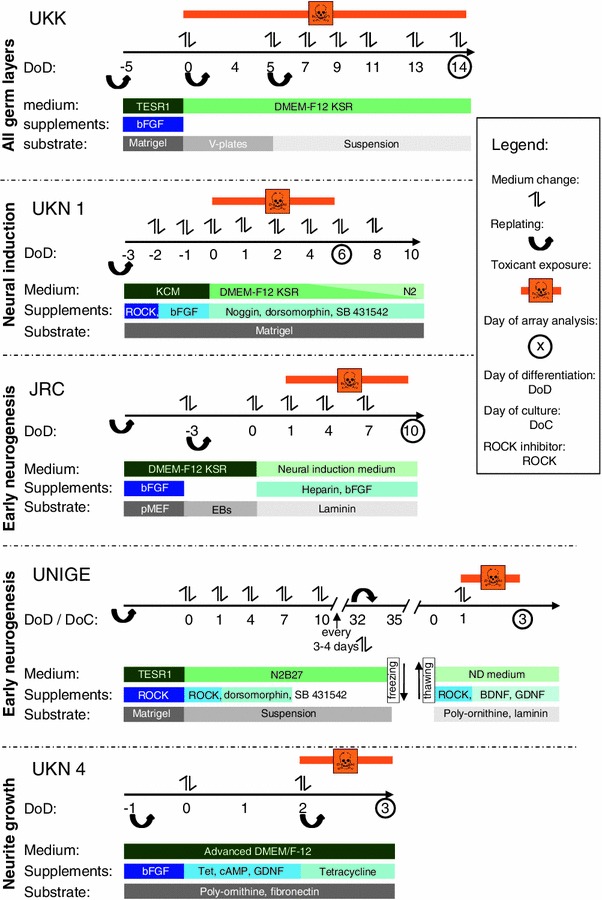
Overview over the test systems’ treatment protocols used for microarray analysis. The five test systems cover different periods and processes relevant to early embryonic/neuronal development, as indicated to the *left*. The *time arrows* indicate when cells were re-plated, medium was exchanged, toxicants were added and when analysis was performed. Additional information is presented below each test system on the type of coating and the medium used in different experimental phases

To bridge this gap, embryonic stem cell (ESC)-based systems are currently being developed (Kuegler et al. 2012; Leist et al. 2008a; Weng et al. 2012; Zimmer et al. 2012). These systems recapitulate early neuronal development in vitro, including neurulation, patterning, neurogenesis and gliogenesis. In the present study, five human ESC (hESC)-based in vitro systems, named here after the developing institutions, have been employed. They recapitulate different phases of early tissue specification and neural development (Fig. 1). UKK recapitulates the multi-lineage differentiation of hESC into ecto-, meso- and endoderm (Jagtap et al. 2011; Meganathan et al. 2012). UKN1 models the stage of neuroectodermal induction that results in the formation of neural ectodermal progenitor cells (NEP) (Balmer et al. 2012; Chambers et al. 2009). JRC reproduces the neural tube formation during early neurogenesis by the formation of neural rosettes (Stummann et al. 2009). UNIGE models the transition from neural precursor cells to mature neurons, showing morphological signs of neural differentiation, including neurite extensions. UKN4 already starts with neuronally committed precursor cells that undergo the maturation towards post-mitotic neurons with neurites. These cells were not derived from hESC but from a human foetal brain (Scholz et al. 2011; Stiegler et al. 2011).

Differentiating murine ESCs show similar waves of gene expression changes as observed during murine embryonic development in vivo (Barberi et al. 2003; Gaspar et al. 2012; Kadereit et al. 2012; Zimmer et al. 2011a, b). Such information is not available for early human development, but it is generally assumed by analogy that hESC would reproduce normal human tissue differentiation (Leist et al. 2008a). Under this condition, transcriptome analysis, including bioinformatic processing of the data, appears as an attractive method to detect perturbations caused by chemicals in the normal wave-like expression patterns in hESC differentiation systems. Moreover, alterations in the proportions of cell types, as a consequence of exposure to test compounds, should be detectable by DNA microarrays (DMA), as shown earlier for other systems (Schmidt et al. 2008, 2012). The treatment period for each test system was chosen according to previously described effects (Fig. 1). For example, in UKN4, neurite outgrowth starts on day of differentiation (DoD) 2 and can be measured at DoD3 (Stiegler et al. 2011). Therefore, DMA analysis was also performed here under similar incubation conditions. In the same vein, it is known for UKN1 that changes in gene expression are best detectable after treatment from DoD 0 to 6 (Balmer et al. 2012) and accordingly transcriptome analysis was done on DoD6 after 6 days of incubation with test compound.

For test system evaluation, we have chosen valproic acid (VPA) and methylmercury (MeHg), two model compounds that trigger RT and DNT in humans and animals (Chen et al. 2007; Grandjean and Landrigan 2006; Kadereit et al. 2012; Wang et al. 2011). The ability of VPA to cause DNT has been recognized since the 1970s. VPA is a clinically used anti-epileptic drug that acts as a reversible modifier of enzyme activities. It has also been shown to cause neural tube defects and to trigger large changes of the cellular transcriptome through the inhibition of histone deacetylases (Jergil et al. 2009; Theunissen et al. 2012a; Werler et al. 2011). MeHg also causes neural tube defects (Grandjean and Herz 2011; Robinson et al. 2011). However, the transcriptional changes due to MeHg are more limited and indirect, as it acts through the unspecific modification of many different proteins, in addition to triggering oxidative stress (Aschner et al. 2007). Despite its unclear mode of action, MeHg is a ‘gold standard’, because human DNT has been particularly well documented, mainly due to the catastrophic endemics caused by MeHg-contaminated food (Bakir et al. 1973; Choi 1989; Davidson et al. 2004; Ekino et al. 2007; Harada 1995).

The widespread use of transcriptomics endpoints requires clarification of important technical issues. Therefore, we addressed here the following questions: (1) Does DMA analysis allow differentiation between distinct classes of toxicants and non-toxicants. If yes, (2) how large is the overlap between the available ESC based test systems (Fig. 1), and are they all required for the identification of DNT compounds? (3) How many independent experiments are needed? (4) At which optimal concentrations should gene array analyses be performed? The present study provides unequivocal answers to these questions and will therefore serve as a basis for further development of RT assays on the basis of DMA classification algorithms.

## Materials and Methods

### Chemicals

Valproic acid (VPA), mannitol and methylmercury chloride (MeHg) were obtained from Sigma. Stocks of VPA and mannitol were prepared in water. MeHg was initially dissolved in 10 % ethanol. A concentration of 10 mM MeHg in this solvent was used as a master stock. For experiments, the MeHg solution was pre-diluted 1:1000 in water (final solvent concentration 0.1 %) and used as the stock for further dilution with medium. The highest test solvent concentration used in this study (at 1.5 μM MeHg) was 0.0015 % ethanol.

### Cell culture maintenance and experimental set-up

#### UKK

NIH-registered H9 human embryonic stem cells (WA09, WiCell Research Institute, Madison, WI, USA) were cultured in DMEM-F12, 20 % KO serum replacement, 1 % non-essential amino acids, penicillin (100 units/ml), streptomycin (100 μg/ml) and 0.1 mM β-mercaptoethanol supplemented with 4 ng/ml human recombinant basic fibroblast growth factor (bFGF) at 37 °C and 5 % CO_2_. The undifferentiated stem cells (hESCs) were routinely passaged with mechanical dissociation on irradiated mouse embryonic fibroblasts (MEF). Prior to differentiation, the cells were maintained for 5 days in 60-mm tissue culture plates (Nunc, Langenselbold, Germany) coated with a hESC-qualified matrix (BD Biosciences, California, USA) in TESR1 medium (Stem Cell Technologies, mTESR1 basal medium + mTESR1 5× supplement). For multilineage differentiation, embryoid bodies (EBs) were prepared as described previously (Jagtap et al. 2011) with minor changes (60–70 clumps were added and bacteriological plates were not coated with pluronic), and the EBs were maintained for 14 days on a horizontal shaker with or without drug treatment. Toxicant exposure was performed as indicated in Fig. 1.

#### UKN1

H9 hESCs (as for UKK) were differentiated by dual SMAD inhibition as described earlier in detail (Balmer et al. 2012; Chambers et al. 2009; Weng et al. 2012). Briefly, hESCs were plated as single cells at a density of 18,000 cells/cm² in medium previously conditioned for 24 h with mitomycin C-inactivated mouse embryonic fibroblasts, containing 10 μM ROCK inhibitor Y-27632 and 10 ng/ml bFGF. Medium was changed daily to conditioned medium containing 10 ng/ml bFGF for 2 days. Differentiation was initiated 3 days after re-plating on day of differentiation (DoD) 0 by changing the medium to knockout serum replacement medium (KSR) (Knockout DMEM with 15 % knockout serum replacement, 2 mM Glutamax, 0.1 mM MEM non-essential amino acids and 50 μM beta-mercaptoethanol) supplemented with 35 ng/ml noggin, 600 nM dorsomorphin and 10 μM SB-431642. From DoD4 onwards, KSR was replaced stepwise with N2 medium (DMEM/F12 medium, 1 % Glutamax, 1.55 mg/ml glucose, 0.1 mg/ml apotransferrin, 25 μg/ml insulin, 100 μM putrescine, 30 nM selenium and 20 nM progesterone), starting with 25 % N2 medium at DoD4. To assess the chemical effects on RNA expression, the cells were differentiated in the presence or absence of the chemicals from DoD 0 for 6 days.

#### JRC

NIH-registered H9 hESCs (WiCell, USA) were cultured undifferentiated in 60-mm cell culture dishes (TPP, Switzerland) at 37 °C and 5 % CO_2_ on a layer of mitomycin C-inactivated primary mouse embryonic fibroblasts (pMEF, CF-1 strain Millipore USA), which were plated at a density of 15000 cells/cm^2^, on gelatine-coated dishes in the presence of the standard maintenance medium for undifferentiated hESCs [DMEM/F12 supplemented with 20 % KO serum replacement, 1 % non-essential amino acids, 2 mM glutamine, 0.1 mM β-mercaptoethanol and 4 ng/ml human recombinant bFGF (all from Invitrogen, USA)]. Cells were expanded weekly by microdissection and further propagated on a feeder layer. For the differentiation towards early neuroepithelial precursors, a published protocol was modified (Stummann et al. 2009). Briefly, intact 6-day-old H9 hESC colonies were detached by 1 mg/ml collagenase (Invitrogen, USA) treatment and left in suspension culture dishes for 3 days in hESC maintenance medium without bFGF to allow the generation of EBs. After this time, EBs were transferred onto single wells (one EB per well) of 96-well plates coated with 10 µg/ml laminin [in water (Sigma, USA)] containing neural induction medium [DMEM/F12 supplemented with 1 % non-essential amino acids, 1 % N2 supplement, 2 µg/ml heparin (Sigma, USA) and 20 ng/ml bFGF (unless stated, all from Invitrogen, USA)]. Cultures were kept for up to 10 days with medium changes every third day. The attached EBs were observed daily and by day 10 they formed neural tube–like structures known as neural rosettes.

#### UNIGE

For neural differentiation, an aliquot of H9 cells (WA09, WiCell Research Institute, Madison, WI, USA) was thawed and cultured in suspension in T75 flasks with N2B27 medium (Life Technologies). From day 2 to 7, cells were incubated in N2B27 medium supplemented with 10 μM anti TGF-beta (Ascent) and 2 μM dorsomorphin (Tocris Bioscience). From day 8 to 32, medium replacement was performed with N2B27 medium only. On day 33, generated spheres were dissociated as single cells and cultured in N2B27 medium in poly ornithine (PLO) and laminin-coated 6-well plates. On day 36, cells were detached and frozen in N2B27 medium in different aliquots. To test neurotoxicity of chemical compounds, an aliquot was thawed in PLO and laminin-coated 6-well plates. Cells were cultured in a neuronal differentiation medium (ND medium) made of NB medium, B-27 supplement, 2 mM l-Glutamine and penicillin/streptomycin (Life Technologies) as well as 10 ng/ml BDNF, 10 ng/ml recombinant human glial cell-derived neurotrophic factor (GDNF) (Chemie Brunschwig) and 10 μM ROCK inhibitor (Ascent). After 1 day of recovery, cells were incubated with the neurotoxicant in ND medium without ROCK inhibitor for 2 days and then material was collected for analysis.

#### UKN4

Lund human mesencephalic cells (LUHMES) were cultured exactly as described earlier (Scholz et al. 2011; Stiegler et al. 2011). Briefly, cells were maintained in advanced DMEM-F12, 1x ‘N2 supplement’, 2 mM l-glutamine and 40 ng/ml bFGF at 37 °C in a humidified 95 % air/5 % CO_2_ atmosphere on Nunclon™ plastic cell culture flasks, coated with 50 ng/ml PLO and 1 μg/ml fibronectin. Proliferating cells were enzymatically dissociated with trypsin (138 mM NaCl, 5.4 mM KCl, 6.9 mM NaHCO_3_, 5.6 mM d-Glucose, 0.54 mM EDTA, 0.5 g/l trypsin from bovine pancreas type-II-S) and passaged every other day.

For differentiation, 8 × 10^6^ LUHMES were seeded into a T175 flask in proliferation medium and differentiation was started after 24 h on day 0 (d0), by changing to advanced DMEM-F12, 1× ‘N2 supplement’, 2 mM l-glutamine, 1 mM dibutyryl 3′,5′-cyclic adenosine monophosphate (cAMP), 1 μg/ml tetracycline and 2 ng/ml GDNF. After 2 days of cultivation in culture flasks, cells were trypsinized and seeded onto PLO/fibronectin-precoated 96-well plates at a cell density of 30 000/well in advanced DMEM-F12, 1× ‘N2 supplement’, 2 mM l-glutamine, 1 μg/ml tetracycline. One hour after re-plating, cells were exposed to toxicants for 24 h.

### Affymetrix gene chip analysis

Analysis was performed as described earlier (Balmer et al. 2012; Jagtap et al. 2011). Briefly, samples from approximately 5 × 10^6^ cells were collected using RNAprotect reagent from Qiagen. The RNA was quantified using a NanoDrop N-1000 spectrophotometer (NanoDrop, Wilmington, DE, USA), and the integrity of RNA was confirmed with a standard sense automated gel electrophoresis system (Experion, Bio-Rad, Hercules, CA, USA). The samples were used for transcriptional profiling when the RNA quality indicator (RQI) number was >8. First-strand cDNA was synthesised from 100 ng total RNA using an oligo-dT primer with an attached T7 promoter sequence, followed by the complementary second strand. The double-stranded cDNA molecule was used for in vitro transcription (IVT, standard Affymetrix procedure) using Genechip 3′ IVT Express Kit. During synthesis of the aRNA (amplified RNA, also commonly referred to as cRNA), a biotinylated nucleotide analogue was incorporated, which serves as a label for the message. After amplification, aRNA was purified with magnetic beads and 15 μg of aRNA was fragmented with fragmentation buffer as per the manufacturer’s instructions. Then, 12.5 μg fragmented aRNA was hybridised with Affymetrix Human Genome U133 plus 2.0 arrays as per the manufacturer’s instructions. The chips were placed in a GeneChip Hybridization Oven-645 for 16 h at 60 rpm and 45 °C. For staining and washing, Affymetrix HWS kits were used on a Genechip Fluidics Station-450. For scanning, the Affymetrix Gene-Chip Scanner-3000-7G was used, and the image and quality control assessments were performed with Affymetrix GCOS software. All reagents and instruments were acquired from Affymetrix (Affymetrix, Santa Clara, CA, USA).The generated CEL files were used for further statistical analysis. The authors declare that microarray data were produced according to MIAME guidelines and will be deposited in ArrayExpress upon acceptance of the manuscript.

### Cytotoxicity testing

In order to determine the cytotoxic range of the chemicals to be tested with the DMA, a resazurin assay was performed in all test systems. The assay is based on the capability of viable and healthy cells to reduce resazurin to resorufin, which can be measured by a colorimetric or fluorimetric shift as described earlier (Stiegler et al. 2011; Stummann et al. 2009). Exposure time to chemicals and day of analyses for this endpoint was the same as for the experimental set-up of the RNA sampling (Fig. 1). Chemicals were tested at several concentrations. Each condition was run in technical triplicates in at least three independent biological experiments. On the day of analysis, cells were incubated with 10 μg/ml resazurin for 30 min to 1 h at 37 °C and 5 % CO_2_. To determine the background fluorescence of resazurin itself, a control with only resazurin in medium was included. Resorufin was measured at a wavelength of 560E×/590Em with a fluorescence reader. The mean background fluorescence of resazurin was subtracted from all experimental data. Further data processing to identify the lowest non-cytotoxic ‘benchmark concentration’ (BMC) of the chemicals was done as follows: data from each experiment were normalised to their respective untreated controls (set as 100 %). The data were then displayed in semilogarithmic plots. Data points were connected by a nonlinear regression sigmoidal dose–response curve fit. These curves were averaged, and the average curve was plotted. The BMC was then determined graphically as the data point on the average curve corresponding to the 90 % viability value, or as the last real data point left of this value. The BMC was used as test concentration for DMA analysis. The ‘lower test concentration’ (LOW) was determined by dividing the BMC by a factor of four.

### In vitro–in vivo extrapolation

In vitro–in vivo extrapolation (IVIVE) of toxicity data can be achieved using physiologically based pharmacokinetic (PBPK) modelling (Carrier et al. 2001; Forsby and Blaauboer 2007; Louisse et al. 2010; Rotroff et al. 2010; Verwei et al. 2006; Wetmore et al. 2012).The extrapolation is based on the implicit assumption that equal concentrations at the target site in vitro and in vivo lead to equal effects. In this project, in vitro nominal concentrations equivalent to relevant toxic concentrations in vivo were determined in two steps. (1) PBPK modelling was used to simulate systemic concentrations corresponding to the lowest dose level at which neurodevelopmental effects were observed in rats. The acslX software was used for the simulations (v3.0.1.6; Aegis Technologies, Huntsville AL, USA). (2) The unbound fraction may differ between in vitro and in vivo systems due to differences in albumin concentrations and lipid fractions between plasma or extracellular fluid and test medium. The nominal in vitro concentration *C*_vitro_ equivalent to the maximum systemic concentration in vivo *C*_pl_ was derived by correcting for these differences by:

$$ C_{\text{vitro}} = C_{\text{pl}} \times \left\{ {\left( {1 - f_{\text{b,pl}} } \right) \times \frac{{1 + K_{\text{ow}} \times {\text{VF}}_{\text{L,vitro}} }}{{1 + K_{\text{ow}} \times {\text{VF}}_{\text{L,pl}} }} + f_{\text{b,pl}} \times \frac{{P_{\text{vitro}} }}{{P_{\text{pl}} }}} \right\} $$

where *f*_b,pl_ is the plasma bound fraction, VF_L,pl_ and VF_L,vitro_ are the volume fractions of lipids in plasma and in vitro, *P*_pl_ and *P*_vitro_ are the concentrations of albumin in plasma and in vitro (Gulden and Seibert 2003). Supplementary figure S6B shows the lipid content and albumin concentrations in the test systems and in rat plasma.

#### IVIVE of MeHg data

The kinetics of MeHg in rats was previously described using a detailed PBPK model by Carrier et al. (2001). This PBPK model was used in the current project to predict systemic concentrations of MeHg after exposure to dosages known to result in relevant toxic effects in vivo. A comprehensive review of neurodevelopmental toxicity of MeHg in laboratory animals was published by Castoldi et al. (2008). The lowest maternal exposures in rat leading to behavioural and neurophysiological effects in the offspring were between 0.01 and 0.05 mg/kg/day from gestation day 6 to 9 (Bornhausen et al. 1980). MeHg extensively binds to intra- and extracellular proteins by the formation of cysteine complexes. The MeHg–cysteine complexes readily pass placental and blood–brain barriers by facilitated transport (Gray 1995). Maternal and foetal blood concentrations were found to be similar (Gray 1995).The total blood concentration was therefore assumed to be available for foetal brain exposure and equated to the nominal concentration in vitro.

#### IVIVE of VPA data

A PBPK model for VPA was developed and calibrated according to data of Binkerd et al. (1988) and Kobayashi et al. (1991). Model equations and parameterization are given in the supplemental material (Fig. S6). This model was used to predict systemic VPA concentrations corresponding to the lowest dose at which neurodevelopmental effects were observed in rats in vivo. A single intraperitoneal dose of VPA in rat dams of 350 mg/kg was found by Rodier et al. (1996) to cause behavioural and neuromorphological effects in the offspring. Oral and intraperitoneal doses lead to comparable plasma kinetics (Ingram et al. 2000). VPA is known to pass the placental barrier in several species; therefore, comparable VPA concentrations were assumed in maternal and cord plasma. The unbound concentration in plasma was equated to the unbound test medium concentrations. For the correction of binding, a bound fraction in plasma of 63 % was used (Loscher 1978).

### Statistical analysis of gene array data

The following analyses were performed using the statistical programming language ‘R-version 2.15.1’ For the normalisation of the entire set of 190 Affymetrix gene expression arrays, the Robust Multi-array Average (RMA) algorithm (Irizarry et al. 2003) was used that applies background correction, log2 transformation, quantile normalisation and a linear model fit to the normalised data to obtain a value for each probe set (PS) on each array. To avoid having to re-normalise future-generated data for comparison with the current data, we used the R package RefPlus (Harbron et al. 2007) that allows the user to perform extrapolation strategies by remembering the normalisation parameters. After normalisation, gene expression for each gene at each concentration was adjusted by comparing the expression to the corresponding control array expression, that is, the difference between gene expressions at each concentration compared to the control was calculated (paired design).

Differential expression was calculated using the R package limma (Smyth et al. 2005). Here, the combined information of the complete set of genes is used by an empirical Bayes adjustment of the variance estimates of single genes. This form of a moderated *t* test is abbreviated here as ‘Limma *t* test’. The resulting *p* values were multiplicity-adjusted to control the false discovery rate (FDR) by the Benjamini–Yekutieli procedure. As a result, for each combination of centre (=test system), compound and concentration, a gene list was obtained, with corresponding estimates for log fold change and *p* values of the Limma *t* test (unadjusted and FDR-adjusted).

### Data display algorithms

General test quality control was as described (Leist et al. 2010). Heatmaps were used to visualise matrices of gene expression values. Colour encodes the magnitude of the values, ranging from yellow (low) to red (high). Volcano plots were used to visualise genome-wide differential expression. Gene wise fold-change values (log2 scale) are plotted against (unadjusted or FDR-adjusted Limma *t* test) significance values (negative log10 scale) on the *x*-axis and *y*-axis, respectively. Principal component analysis (PCA) plots were used to visualise expression data in two dimensions, representing the first two principal components, that is, the two orthogonal directions of the data with highest variance. The percentages of the variances covered are indicated in the figures. The software ‘R - version 2.15.1’ was used for all calculations and display of PCA and heatmaps (R_Development_Core_Team 2011). The calculation and display of toxicity curves was done using GraphPad Prism 5.0 (Graphpad Software, La Jolla, USA). The Venn diagrams for the comparison of gene expression, gene ontology (GO) terms and transcription factor binding sites (TFBS) between test systems were constructed according to Chow and Rodgers (2005). The size of circles and areas was chosen proportional to the number of elements included.

Transcription factor binding site enrichment (TFBSE) was performed using the PRIMA algorithm (Elkon et al. 2003; http://acgt.cs.tau.ac.il/prima/) provided in the Expander software suite (version 6.04, (Ulitsky et al. 2010); http://acgt.cs.tau.ac.il/expander/). Lists of significant differentially expressed genes with adjusted *p* value <0.05 were converted to Entrez IDs (R package hgu133plus2.db) and duplicates were removed. The PRIMA algorithm was run with a *p* value threshold set to 0.05, no multiple testing correction, a background set of all human genes (provided in the Expander software suite), and using the TRANSFAC database (8.2) as the data source for transcription factor binding sites. The PRIMA algorithm analyses 267 separate TRANSFAC entries. PRIMA results are presented in tables with TF identifiers provided by PRIMA and their full names, or the overlap between TF enrichments for different treatments is shown as Venn diagrams or as tables [Cytoscape; (Shannon et al. 2003; Smoot et al. 2011); http://www.cytoscape.org].

For the word clouds of the overrepresented GO groups, a g:Profiler query (Reimand et al. 2007) was initially made, and only results from the biological process and pathway branches were retained. These were viewed as a subgraph of the whole GO tree. All categories were deleted that were larger than 1,000 genes and smaller than 50 genes. Then, connected components from the remaining graph were identified, and from each of these, the category with the highest *p* value was selected. These were ordered by *p* value and the top 40 are displayed. When displaying the categories, the font sizes were first scaled to be proportional to the log10 of enrichment *p* value. To enable global comparison, the grey shade of the letters was scaled the same way over all plotting windows.

To assess the sensitivity of differential expression analysis with respect to the number of DMA (=experimental replicates), the following approach was used: For each condition, we identified the differentially expressed genes based on five pairs of DMA (control vs treated), which was then used as the reference list. Significant PS were identified in all cases by Limma *t* test, with a *p* < 0.05 as significance threshold. The Benjamini–Hochberg and the Benjamini–Yekutieli were used for the FDR correction in different experiments as appropriate and as specified in the figure legends. All possible permutations of 2, 3 or 4 DMA were calculated, and the differentially expressed PS of all these conditions were identified (using the same method as for the reference calculation). Finally, the overlap between the new gene lists and the reference was calculated, to determine the quantity of the reference that could be recovered with less DMA.

## Results and discussion

### Detection of different transcriptional responses to the DNT model compounds, valproic acid and methylmercury

To explore the dynamics and specificity of the transcriptional response of novel hESC-based in vitro systems (Fig. 1), we chose VPA and MeHg as two positive control toxicants with described effects on DNT and D-mannitol as the negative control compound. The three test compounds were initially evaluated in three of the test systems (UKK, UKN1 and JRC) at the ‘maximum tolerated concentration’. This benchmark concentration (BMC) was determined experimentally for each of the test systems as the highest concentration that reduced overall cell viability by not more than 10 % (Fig. S1). In the case of mannitol, a large range of concentrations, from 1 μM to 100 mM, was used and no cytotoxicity was detected (data not shown). For the UKN1 system, the response to mannitol was tested by quantitative PCR for three toxicant-responsive genes (*OCT4*, *Pax6* and *OTX2*) (data not shown). As no changes were observed for concentrations up to 40 mM, and data on this compound were provided by the other test systems, DMSO (28 µM) was chosen as the DMA-negative control for UKN1. The transcriptional alterations triggered by the BMC of the two toxicants (VPA/MeHg) or by the two negative controls (mannitol/DMSO) were measured in 4–5 independent experiments on Affymetrix DMA, and the genes that were differentially expressed between culture medium-only controls and test compounds were determined by modern stringent statistical methods (Limma *t* test, Benjamini–Yekutieli FDR correction). The complete set of data is displayed in supplementary Table S1.

**Fig. 2:**
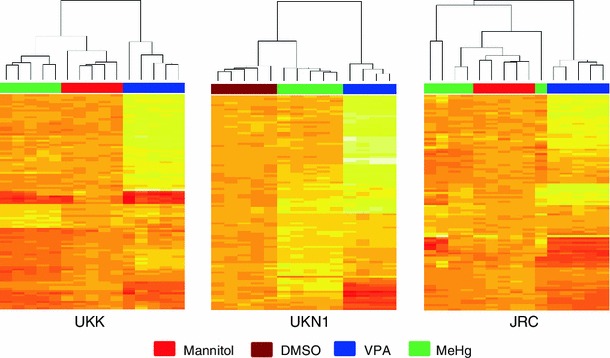
Differential alterations of gene expression by valproic acid (VPA) and methylmercury (MeHg). Three different test systems (UKK, UKN1 and JRC) were exposed to VPA (*blue label* on top of the heatmap) or MeHg (*green label*) at their respective benchmark concentration, or to D-mannitol (*red*) or DMSO (*dark red*). The differentially expressed genes (vs untreated controls) were determined in 4–5 independent experiments (shown as columns of the heatmaps). The similarity of the gene expression patterns is indicated by the Pearson’s distance dendrogram at the *top*. The heatmaps are based on 100 selected genes. These comprise the 50 genes with the lowest adjusted *p* values according to the Limma *t* test for regulation by MeHg, and 50 genes with the lowest adjusted *p* values for VPA. The *colours* of the heatmap indicate the relative gene regulation level above (*red*) or below (*yellow*) the average for each row

For a visual monitoring of the different compound effects, the hundred most regulated (defined by the lowest FDR-corrected *p* values) genes (top 50 for VPA and top 50 for MeHg) were selected for each test system (Table S1), and their relative expression levels were displayed as heat maps. For all test systems, striking differences were observed between the regulation patterns of VPA and MeHg. Clustering analysis showed that VPA samples were clearly separated from the MeHg samples (Fig. 2). This effect was even more pronounced when clustering was performed with the 100 top genes regulated by VPA (Fig. S2A). Under these conditions, the differences between MeHg and negative controls were small or not apparent. Therefore, clustering was also performed with the top 100 genes regulated by MeHg. Under these conditions, MeHg samples were clearly separated from those treated with D-mannitol/DMSO (Fig. S2B).

**Fig. 3:**
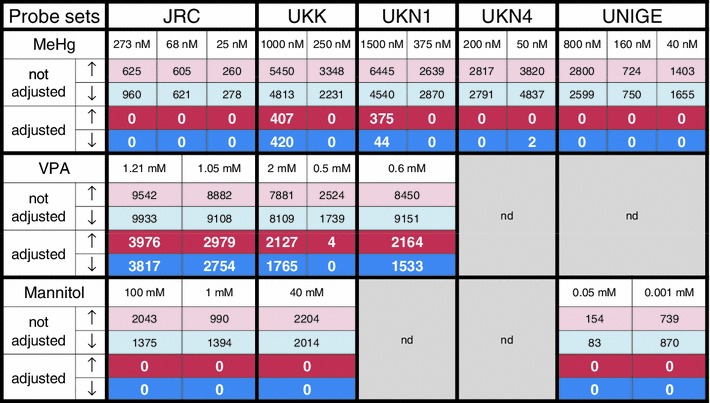
Overview of differentially expressed genes in all test systems. Positive and negative control compounds were tested in the JRC, UKK, UKN1, UKN4 and UNIGE test systems. The test concentrations for methylmercury (MeHg), valproic acid (VPA) and D-mannitol (Mannitol) are indicated in the *white fields*. The number of significantly altered probe sets (PS) is indicated separately for up-regulations (*red*) and down-regulations (*blue*). The results for testing without FDR adjustment are indicated in pale-coloured fields. The results after FDR adjustment by the Benjamini–Yekutieli method are indicated in *white bold numbers*. The highest compound concentration tested corresponded to the BMC of the respective test system. The highest test concentration (800 nM) was five times higher than the BMC (160 nM) for UNIGE only. *nd* not done

The number of significantly altered Affymetrix DMA probe sets (PS) was much higher for VPA compared to MeHg. The sum of all PS changed by VPA in the test systems UKK, UKN1 and JRC was 15386; for MeHg, the sum was 1246 PS (Table S1, Fig. 3). This striking difference was observed, although both compounds were used at their respective BMC in each test system. Exposure to the negative controls did not result in any significant changes (Fig. 3). Thus, the extent of the responses of the neurally differentiating hESC to the different developmental neurotoxicants appears to be compound-specific. Moreover, the responses to the two model toxicants differed qualitatively (Fig. 2; Fig. S2). The ability to clearly distinguish known toxicants suggests that the test systems would distinguish unknown classes of potential toxicants. It may be speculated that safety liabilities of unknown chemicals for humans may be predicted by comparing their effects in the test systems with those of known toxicants and non-toxicants. The technical and statistical basis of the above initial findings, together with their potential biological and toxicological implications was explored further in the following extended test battery.

### Differential constitutive and toxicant-induced responses of the test battery

One may hypothesise that MeHg showed only relatively weak effects in the initial testing (UKK, UKN1 and JRC) as all these systems only generate immature cells, and such cells may be relatively resistant to MeHg. Alternatively, such test systems may lack key targets of mercury toxicity. Such an assumption would be in agreement with findings in neuronally differentiating murine ESC, which were highly sensitive to MeHg during the late neuronal maturation phase, but relatively insensitive during the initial phase of neural precursor formation (Zimmer et al. 2011b). For a broader coverage of effects during later phases of neurogenesis, two additional test systems were used (Fig. 1, UNIGE and UKN4). The UNIGE hESC-based test system covers the developmental phase after neural stem cell formation. The UKN4 test system was used as reference, as this system is well characterised not only for transcriptome changes, but in particular for functional and phenotypic effects (Stiegler et al. 2011). From the literature, it is known that MeHg inhibits neurite outgrowth in this system, and transcriptome analysis was performed at a concentration known from previous studies to affect neurites (Stiegler et al. 2011).

The extended test battery (UKK, JRC, UKN1, UKN4 and UNIGE) was used for additional testing. The effects of MeHg were examined in all systems at the respective BMC, in addition to one lower concentration (LOW). The latter was determined by dividing the BMC by a factor of four (Fig. S1). Additional experiments were also performed with VPA. The compound was tested at two relatively similar concentrations in JRC (to test the reproducibility of the response). It was also examined at fourfold different concentrations in UKK (to test potential concentration dependencies of the response). The number of differentially expressed PS for each condition is summarised in Fig. 3. This broad experimental approach showed that the transcriptional response of differentiating hESC to MeHg is indeed very limited. Also, the test systems using more mature cells (UKN4 and UNIGE) did not show any significant response when stringent FDR corrections were used.

**Fig. 4:**
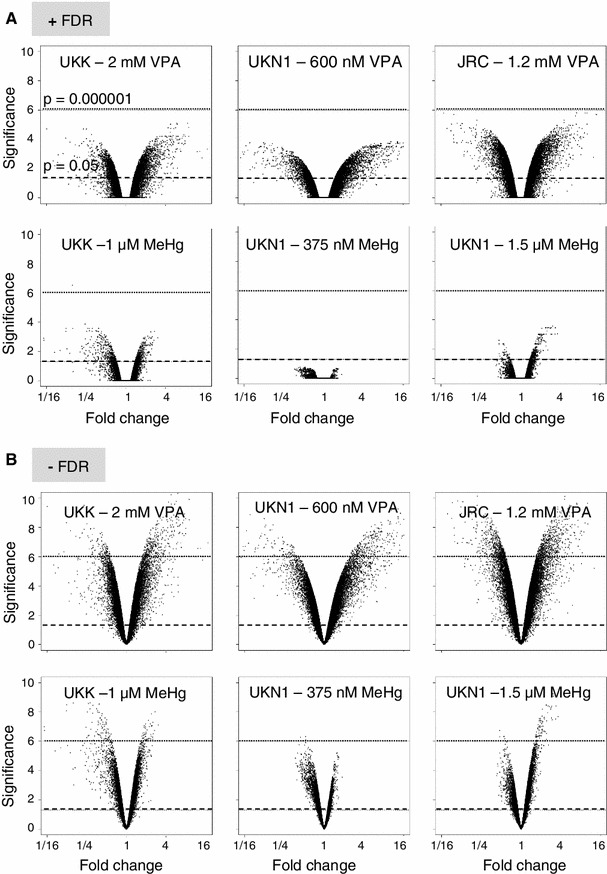
Correlation of fold change and significance level of gene expression for different statistical approaches. Data were generated and calculated for each combination of test system and compound, as illustrated in Fig. 3. In the volcano plot diagrams, fold changes of the compound-induced gene expression are shown on the *x*-axis (log2-scale). The *y*-axis shows negative logarithmic-adjusted *p* values of a LIMMA *t* test (−log_10_(*p* value)). The *p* values were **a** FDR-adjusted, or **b** not FDR-adjusted. The *dashed lines* show the significance level of *p* = 0.05. The *dotted lines* show an example for the *p* = 0.000001 significance level for orientation. All other test systems and compounds are shown in the supplemental material (Fig. S3)

**Fig. 5:**
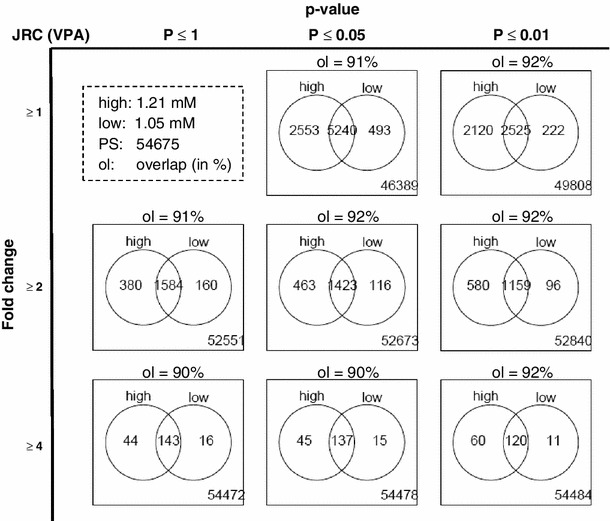
Overlap of differentially expressed probe sets (PS) at different concentrations. The JRC test system was exposed to VPA at a high (=BMC) and low concentration in five independent experiments. The *circles* of the Venn diagrams show the numbers of PS that were influenced by the two experimental conditions. The overlap gives the number of genes influenced both at the low and the high concentration. The fraction of the genes in the overlap (ol) with respect to all genes altered at the low concentration is indicated above each diagram. The number at the *lower right corners* indicates the number of PS not influenced by the test compound at any concentration. Significance was determined by the LIMMA FDR-adjusted *t* test. The *first column* shows results without restriction by the *p* value and examines the effect of restrictions by the fold-change value on the number of PS identified. The *second column* imposes the additional restriction that all identified PS should have a *p* value below 0.05. The *third column* shows the results when only PS with a *p* value below 0.01 are selected

Comparison of the results before and after FDR correction showed the unmistakeable need for appropriate statistical treatment of the data. Although the choice of a 5 % significance level will generate on average 2734 false positives when 54675 PS are analysed (as in this study), it can at times still be counter-intuitive for toxicologists when none of the more than 2000 identified genes is significant after FDR correction. The effect of FDR correction in the present study is visualized in the form of volcano plots. This form of display orthogonally separates the two parameters usually considered important in gene expression analysis: the fold change and the significance level. As the FDR correction only affects the significance level, one can see the ‘volcano’ heights being compressed, while the width remains the same; for instance, in the case of JRC incubated with 273 nM MeHg (BMC), all apparently significant PS dropped below the usual significance level (*p* < 0.05). Also, with UKK exposed to 500 μM VPA (20 % of the BMC), the number of 2524 PS that appeared to be significantly up-regulated before FDR correction dropped down to four really significant PS after FDR correction. Notably, the apparent significances were ‘lost’, although several PS appeared to be ‘regulated’ more than twofold, at times even up to fourfold (Fig. 4, Fig. S3). It should be noted that the gene expression response occurred within a narrow range of concentrations. The FDR-corrected data sets showed that the number of regulated probe sets can change from several thousands to zero within a fourfold concentration range. Even a lowering of the test concentration by only 20 % (relative to the BMC) resulted in a reduction of the identified PS, at least in one system in which this was tested (JRC). However, more than 90 % of the PS identified at the low concentration in this assay were also identified at the high concentration (Fig. 5). This good overlap confirmed a robust and reproducible test system response. When more stringent conditions were used for filtering, such as the requirement for a ≥4-fold change or for a lower *p* value, the good overlap between the two concentrations was maintained (Fig. 5). Altogether, these data suggest that the most pronounced and robust transcriptional responses can be measured at toxicant concentrations, which are close to or at the BMC.

**Fig. 6:**
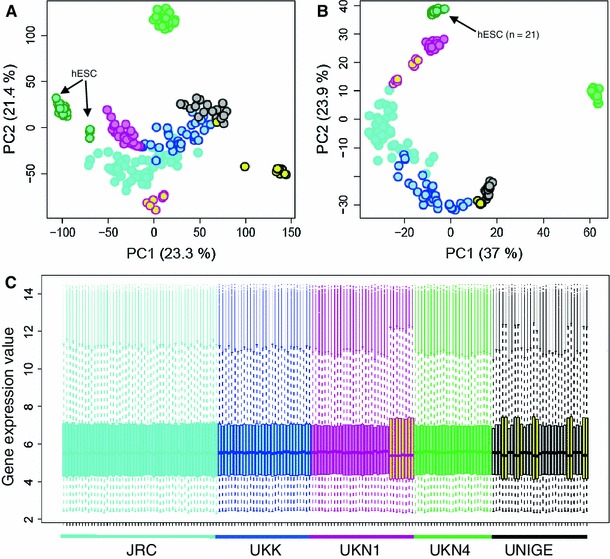
Identification and correction of DNA microarray (DMA) batch effects. The signal of all PS was determined in five different test systems after incubation with compounds as in Fig. 3. The data for every experiment *plus* those of 25 untreated controls and solvent controls and 21 samples of untreated hESC (*dark green circles* with *light blue filling*) were used for principal component analyses (PCA) of altogether 190 DMA. Data from the different test systems are colour-coded, and each DMA is displayed as a circle in the PCA plot. *Circles filled in yellow* code for DMA that clustered away from their respective main groups, and that were considered outliers due to a batch effect, as they were measured at another time point compared to the other samples. The axis labels indicate the percentage of the total variance covered by the respective axis **a** The PCA is based on all PS. **b** The PCA is based only on the 500 probe sets with the highest variance. **c** The distribution of the PS fluorescence signals (indicated here as ‘gene expression value’) is displayed for all 169 test system DMA of this study (each DMA is represented by *one box* of the *box plot*). The size of the boxes indicates the 25th and 75th percentile (the lower and upper quartiles, respectively) of the PS. The *solid lines* in the box indicate the 50th quantile of the distribution. The height of the box being equal to the difference between the upper and lower quartiles is called the interquartile range (IQR). The *dashed lines* (*whiskers*) indicate gene expression values within the range of 1.5 IQR from the 25th and 75th percentile. The *dots* outside the *dashed lines* (appearing as *solid line* due to the print resolution) represent the outliers within one DMA. The DMA corresponding to the differently clustering samples in **a** is indicated by boxes filled with *yellow*, and they show a higher variance. The test system colour coding of part a, b and c is identical

**Fig. 7:**
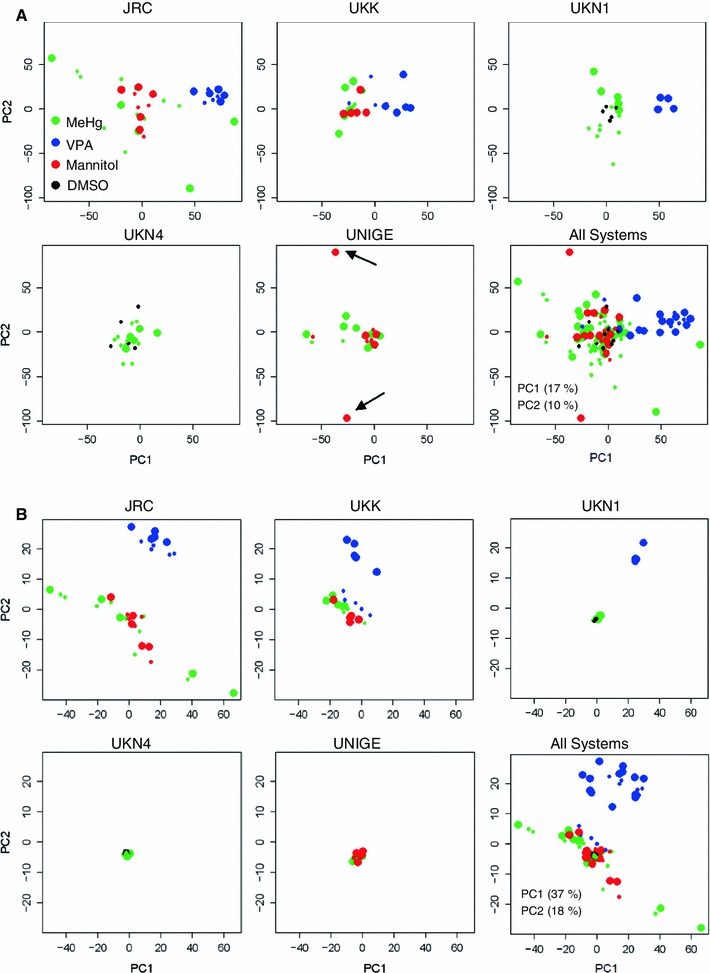
Principal component analysis (PCA) of relative gene expression data after subtraction of solvent controls. **a** The signal of all PS was determined in five different test systems (UKK, UKN1, JRC, UKN4 and UNIGE) after incubation with compounds as in Fig. 3. Then, the values for the respective controls were subtracted from the values of the DMA treated with VPA at the BMC (*large blue*) or at the LOW concentration (*small blue dots*), or MeHg (*large and small green dots*), or D-mannitol (*red*), or DMSO (*black*). These data were then used for PCA. The lower right panel shows all data together. The other panels show the data for individual test systems within the same axes as for all systems. In **a,** all PS were included, while in **b,** only the 500 PS with the highest variance were used. Note for instances, the outliers in UNIGE marked by *arrows* in a, and their perfect clustering in b

To obtain a better overview of how the different test systems are related to one another, we performed a principal component analysis (PCA) encompassing untreated controls and non-differentiated H9 hESC, in addition to all treated samples. This approach allowed the visualization of the overall transcript patterns measured by 190 DMA on a 2-dimensional PCA space (Fig. 6a). Several conclusions can be drawn from a qualitative analysis of the PCA presentation: First, all test systems clearly differed from non-differentiated hESC. Second, all test systems differed from one another, that is, the variance between the different test systems was larger than the variance of individual samples within a given test system. Third, samples from one test system clustered together, whether they had been treated with VPA, MeHg or solvent. On the other hand, samples treated, for example, with MeHg in different test systems did not cluster together in this form of data presentation. It is noteworthy, that presentation of data in the form of such a comprehensive PCA does not allow the identification of compound effects, although large, statistically significant transcriptome changes occurred (e.g. VPA vs solvent control). To better visualise compound effects, a different statistical treatment is required before the data are presented; for instance, the large influence of the different test systems can be attenuated by the subtraction of the corresponding controls before display (see below and Fig. 7).

The distinct clustering of all test systems to a different area of the PCA plot suggests that the test battery is not redundant. Each individual test system seems to react with different transcriptome changes, and the combination of the tests may thus provide richer data than any individual test. This would imply that the different systems would be able to identify different toxicant effects and thus be complementary in their toxicological information. The test battery may thus constitute an important step towards the replacement of animal tests by information-rich human cell-based models (Hartung and Leist 2008; Leist et al. 2008b). This will, however, require further testing and validation (Leist et al. 2012). A second important observation was the presence of outliers in some samples, which will be investigated in greater detail in the following section (Fig. 6a).

### Control of intra-group variability and batch effects

The PCA indicated that eight of the DMA of UKN1 clustered separately from all other UKN1 samples. The commonality amongst the eight DMA was that they were measured on a different day compared to the other samples. Four corresponded to controls and four to samples treated with VPA. Thus, the clustering was not treatment-related. A similar situation was observed for ten samples of UNIGE (Fig. 6a). When only the 500 probe sets with the highest variance were considered for the PCA, the ‘outliers’ moved partially or completely back, that is, they clustered together with the other samples within their test system (Fig. 6b). This suggested that genes with a low variance had contributed to the outlier effect. A graphical presentation of the variances of all DMA performed for this study indeed indicated that the ‘outliers’ had a higher variance of the fluorescence signals, although the average signals were quite similar to all other DMA (Fig. 6c). These data suggest that the ‘distant clustering’ samples are the consequence of a batch effect.

The presented study is still ongoing and even larger numbers of samples will have to be studied. This makes it impossible to analyse all samples in a single batch. Methods to control for batch effects will therefore be required. As indicated here, one possibility is to include only the PS with highest variability between the samples into the analysis. As an alternative approach, the corresponding control values were subtracted from the compound-treated samples before the PCA. This form of presentation clearly separated VPA and MeHg incubated samples, and the results obtained by clustering analysis within the individual test systems were confirmed, also when this multi-systems approach was chosen (Fig. 7a). The subtraction of the controls resulted in the visualization of treatment effects in the PCA that were not visible when the non-processed data were used (Fig. 6). When only the 500 PS with the highest variance—rather than all 54,575 PS—were included, there was a more defined clustering of the VPA samples compared to the MeHg samples (Fig. 7b). The reduction to 500 PS also resulted in a better clustering of other ‘distant clustering’ samples. A stepwise reduction of PS showed that 500 PS seems to represent a reasonable choice, although even smaller numbers, for example, 200 PS, would be possible (Fig S4). An interesting implication of this observation is that the scattering of samples within one group can be caused by relatively large numbers of PS with low variability and not necessarily by the PS which show the highest variance. These ‘high variance PS’ appear to be highly relevant for further analysis.

### Robustness analysis: role of the number of biological replicates

**Fig. 8:**
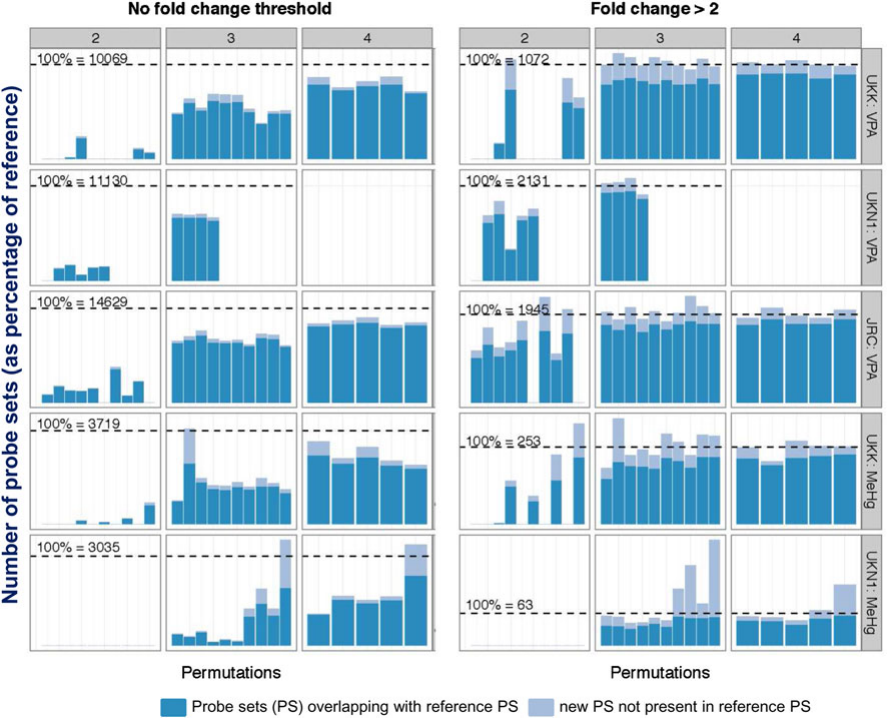
Simulation of different numbers of experiments (pairs of DMA) and their impact on the numbers of significantly regulated PS. VPA was tested in the test systems JRC and UKK at its BMC in five independent experiments, and in UKN1 in four experiments. MeHg was tested in UKN1 and UKK in five experiments. The number of significantly regulated genes (Benjamini–Hochberg FDR correction) was calculated without further restrictions (*left*) or with the restrictions that the PS should be regulated more than twofold (*right*). The numbers of PS are indicated above the dashed black lines, which were set as 100 % reference points. The *dark blue bars* indicate how many of these PS were identified when different permutations of 2, 3 or 4 experiments (indicated as *grey headings*) were used. The *light blue bars* indicate how many additional PS were identified when only subsets of the original five (4) experiments were analysed; for instance, the *five bars* in the panel with the coordinates 4/JRC:VPA represent the five possible ways of omitting one of the experiments. The *10 bars* in the panel with the coordinates 3/JRC: VPA represent the 10 possible permutations of leaving out two of the experiments and then recalculating the significant PS on the basis of the remaining 3 DMA

In the present study, five biological replicates (independent experiments performed at different days) were generated for most test conditions. One technical replicate (one DMA) was analysed per experiment. To study whether lower numbers of DMA would also lead to similar results in the present data set, we chose a statistical permutation approach that simulated the situation of choosing only 2, 3 or 4 of the 5 experimental replicates (Note that each replicate consisted of a matched pair of DMA for control and for treated cells). For each possible combination of these pairs (here for simplicity called DMA or replicates), the number of PS that overlapped with the original set of PS was identified. In addition, new PS that had not been originally identified were also detected. The expectation was that whether 5 DMAs were redundant, then the percentage of original PS identified with 3 or 4 DMA should also be high, and the number of new PS arising from the new analysis should be low. This approach was run under different conditions. The significant genes were identified by the less stringent Benjamini–Hochberg FDR correction (Fig. 8) or by the very stringent Benjamini–Yekutieli correction (Fig. S5). Moreover, either all PS were considered, or only the ones regulated more than twofold (Fig. 8, Fig. S5).

The results showed that there was only a moderate advantage of using 5 DMA instead of 4 when only PS with ≥2-fold changes were considered in the current data set. Under this condition, and using less stringent FDR correction, even 3 DMA would have resulted in the identification of a large majority of genes. The permutation analysis was also found to be a suitable tool to test data consistency and robustness of the analysis method used. For most test systems, removal of any of the 5 DMA (pairs) to generate a new data set based on 4 DMA yielded largely similar results. This suggests that all different experiments had generated largely similar data, although they were performed with different cell cultures on different days. The situation was different for the MeHg samples from UKN1, where removal of one specific DMA resulted in the identification of more than twice as many significant PS compared to the remaining 4 DMA. All combinations of the three remaining DMA that lacked the apparent ‘outlier’ identified much larger numbers of PS compared to the combinations that included that specific DMA (pair) (Fig. 8). Such an analysis may therefore be used to develop statistical techniques for the identification of outliers.

### The relationship between cytotoxic response and DNT-specific transcriptome changes

The choice of toxicant concentrations for gene expression analysis is a critical step. If too high concentrations are used, cell viability will be compromised. The cell death occurring under these conditions may result in unspecific ‘toxicity-associated’ gene expression responses. Conversely, the use of too low concentrations of test compounds would result in false-negative responses and in the inability to identify any alterations of the transcriptome. The magnitude of the response may be dependent on the concentration of the test compound, which is especially important when compounds are compared and possibly classified or ranked according to their specific responses. Furthermore, information on the concentration dependence may be used for more detailed characterisation of compound effects, and possibly for the identification of the hazardous responses as opposed to counter-regulations and unspecific responses (Theunissen et al. 2012a, b).

In the present study, the BMC of the cytotoxicity test (i.e. the highest non-cytotoxic concentration) was used as the standard test concentration (Fig S1). Although transcriptional responses can be triggered by MeHg and VPA at concentrations considerably lower than the cytotoxic concentration (Balmer et al. 2012; Zimmer et al. 2011b), we found here that the majority of responses to MeHg in UKN1 was lost even at only fourfold lower concentrations than the BMC. We made similar observations for VPA in other test systems.

In in vivo studies, DNT is defined as effects on the pups in the absence of maternal toxicity. A corresponding definition for in vitro test systems would be ‘specific alterations of differentiation in the absence of overt (unspecific) cytotoxicity’. Fulfilment of this condition was carefully explored, and several features of our data indicate that measurements at the BMC do in fact allow us to draw conclusions on DNT-specific disturbances triggered by the test compounds: First, we tested whether known toxic concentrations (800 nM MeHg in UNIGE; BMC was 160 nM) would lead to unspecific transcriptional responses (Fig. 3). Also under this condition, no significant PS were identified, that is, no cell death genes were triggered. We also examined the effect of accidental variations of the cytotoxicity from experiment to experiment. The fixed BMC indicated here was determined from a set of pilot experiments. However, the actual cytotoxicity in the individual experiments in which mRNA levels were analysed showed some biological variation, which was documented, for example, for UKN1 and UKN4. Examination of these data showed that the MeHg concentration used for UKN4 reduced cell viability more than the one used for UKN1. However, no response was observed in UKN4, while an apparently specific response was triggered in UKN1. Second, some concentrations used for testing VPA in UKN1 triggered toxicities of more than 10 % (data not shown) in the experiments used for DMA analysis (due to daily experimental variations in sensitivity), but cell death-related GO terms were not identified. In contrast, MeHg in the same system did not trigger measurable cytotoxicity, but GO term analysis indicated an up-regulation of genes related to apoptosis and neuronal death. Thus, the use of compounds at the BMC does not seem to be problematic. In the case of MeHg, triggering of cytotoxic responses is rather a specific feature of the compound (protein modifier, trigger of oxidative stress). This may be an explanation for the low or absent transcriptional responses in the test systems. Third, candidate genes typically related to cell death, DNA damage and oxidative stress were examined in UKN1. Such genes were not overrepresented amongst the VPA-regulated genes. Moreover, their extent of regulation did not correlate with the overall magnitude of regulation in the individual experiments (not shown). Fourth, it was examined how far the responses to different toxicants overlapped. In case of a strong component of cytotoxicity, it was expected that typical stress genes were induced and similarities would be observed in the regulation pattern of different toxicants. However, only a small fraction of the overall altered PS overlapped between VPA and MeHg [as examined in detail below, (Fig. 10)]. Even though a ‘common transcription factor response’ between VPA and MeHg of 16 transcription factors (TFs) was observed, there was still a majority of TFs unique for MeHg or VPA. Thus, two compounds, both used at the BMC, triggered different responses, with no common cytotoxicity pattern.

In summary, the data indicate that the measurement of transcriptional responses at the BMC is a reasonable approach, although further studies are required for a better understanding of a possible ‘common toxicity-associated response’. Our limited set of data indicates that concentrations beyond the BMC do not necessarily result in an unspecific transcriptional response reflecting cytotoxicity.

### Relationship of the BMC with respect to the in vivo relevant concentration range

**Fig. 9:**
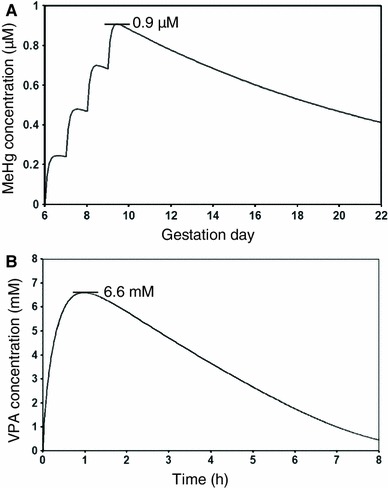
Physiologically based pharmacokinetic (PBPK) modelling of the positive control compounds MeHg and VPA. Systemic concentrations of MeHg (total blood concentration, *upper panel*) and VPA (plasma concentration, *lower panel*) in rats following exposure to a developmental neurotoxic dose predicted by PBPK modelling. **a** PBPK simulation of MeHg total blood concentration in rat dams upon daily oral gavage of 4 mg/kg MeHg on gestation days 6–9, the lowest developmentally neurotoxic dose in Bornhausen et al. (1980). Predicted maximum total blood concentration of 0.9 μM is indicated. Maternal and foetal blood concentrations are considered similar. The foetal total blood concentration is assumed to be available for foetal brain exposure and equated to the nominal concentration in in vitro test media. **b** PBPK simulation of VPA plasma concentration in rat dams upon a bolus intraperitoneal dose of 350 mg/kg, the lowest dose causing relevant effects in Rodier et al. (1996), resulting in a predicted maximum total blood concentration of 6.6 mM (as indicated). Comparable concentrations have been found in maternal and foetal plasma. The unbound plasma concentration in vivo is equated to the unbound concentration in in vitro test media

Besides the technical considerations concerning the BMC and cytotoxicity, the relevance of the chosen concentrations for the in vivo conditions needs to be considered. When in vitro concentrations differ by more than one order of magnitude from concentrations causing toxicity in vivo, pathways of toxicity may become activated that are not relevant to the in vivo situation. Unfortunately, human exposure measurements of DNT compounds are often poorly documented and concentrations in the brain are only rarely known. Nevertheless, human relevant concentrations of 0.005–0.5 μM MeHg and 500–1,000 μM VPA have been reported in a recently published review (Kadereit et al. 2012). To obtain a clearer picture, we used physiology-based pharmacokinetic (PBPK) modelling to calculate in vivo relevant blood and brain concentrations from the doses that caused DNT in animal studies (Fig. 9; Fig. S6A). Oral exposure to MeHg of 0.01 mg/kg on gestation days 6–9 is predicted to result in a maximum total blood concentration of 0.9 μM (Fig. 9a). Thus, similar nominal concentrations should show activity in vitro, although the actual amount of MeHg penetrating the cells may additionally depend on cysteine concentrations in the different media of the test systems. A VPA plasma peak concentration of 6.6 mM is predicted after a single oral dose of 350 mg/kg. This dose resulted in the same model in DNT (Rodier et al. 1996) (Fig. 9b). For extrapolation of such data to in vitro systems, corrections for differences in protein binding and lipid partitioning in plasma vs cell culture medium have to be considered (Fig. S6B). Our calculations suggest that the expected equivalent nominal concentrations in vitro are 3.3 mM for UKK, 2.7 mM for UKN1 and 0.9 mM for JRC, UKN4 and UNIGE. These results show that the BMC concentrations used in this study are within the same order of magnitude as the in vivo concentrations which caused DNT in humans and animals.

### Remarkable overlap of overrepresented TFBS amongst genes influenced by VPA and MeHg

The main focus of this study was to investigate the technical feasibility of using transcriptomics as a major endpoint to characterise responses of hESC-based test systems. For a detailed characterisation of the biological responses of the test systems to the compounds, a different experimental design would be required. Nevertheless, we performed some initial comparisons of gene ontologies (GO) and transcription factor binding sites (TFBS) that were overrepresented amongst the regulated PS. The main aim was to find out whether simple analysis tools can reveal differences and commonalities of the transcriptome responses.

For this approach, five sets of data were compared: the responses of UKN1, JRC and UKK to VPA and the responses of UKN1 and UKK to MeHg (all at BMC concentration). To obtain an overview over the main biological processes affected by co-regulated genes, the statistically overrepresented GO terms were identified and displayed for each test system and condition (Fig. S7); for instance, the genes down-regulated in each test system by VPA pointed to effects of the toxicant on RNA processing, and on chromatin modification/histone acetylation. The latter results are consistent with the known activity of the compound as a histone deacetylase inhibitor (HDACi). GO terms related to effects on ‘neural tube formation’ ‘neuron development’ and ‘embryonic morphogenesis’ showed up for different conditions. These findings gave a hint that there may be an overlap of higher order biological responses across the test systems and compounds. However, we are aware of the fact that the GO term analysis is a very rough tool, and that GO term annotations of many genes can be problematic (Weng et al. 2012). Therefore, we chose the alternative approach of comparing the overlap of regulated PS between the test systems with the overrepresentation of 267 human TFBS (as indirect indicator of higher order linked biological processes).

**Fig. 10:**
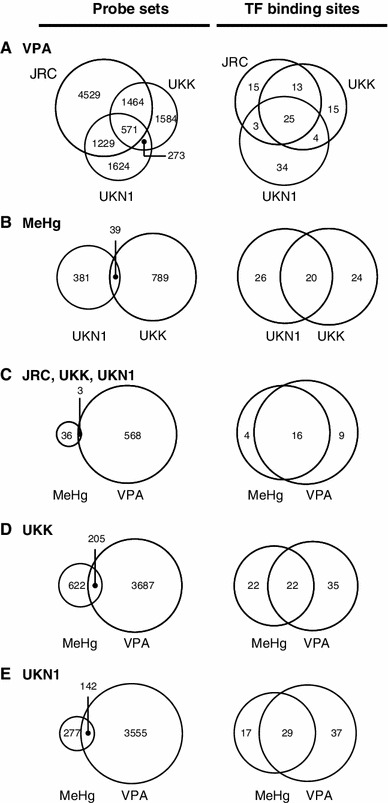
Overlap of altered genes and of overrepresented transcription factor (TF) binding sites between test conditions. Five sets of data, as described in Fig. 3 were used for further analysis and comparisons: exposure of UKK and UKN1 to both VPA and MeHg and of JRC to VPA. All toxicants were used at their BMC. The numbers of differentially expressed probe sets (Limma *t* test, Benjamin–Yekutieli-adjusted *p* value <0.05), and enriched transcription factor (TF) binding sites (PRIMA, *p* value <0.05) were identified. The data are presented as pairs of Venn diagrams, with PS to the *left* and TFBS to the *right*. *Numbers* on the diagrams show the relevant count for each sector of the diagram. The following sets of data are compared: **a** responses to VPA treatment in the JRC, UKK and UKN1 test systems; **b** responses to MeHg treatment in UKK and UKN1 (N.B. for display rules: 44 TFBS were changed in UKK, 20 of which overlapped with UKN1); **c** the circles marked ‘VPA’ show the number of PS/TFBS regulated in all three test systems by VPA, the circles marked ‘MeHg’ show the number of features co-regulated in UKN1 and UKK by MeHg; **d** responses of UKK alone to MeHg or VPA; **e** responses of UKN1 to MeHg and VPA

First, the overlap of test systems treated with the same compound was analysed. VPA regulated 571 PS in all three test systems (Fig. 10a). Thus, only a relatively minor overlap occurred on the level of individual PS. The PS for VPA showed enrichment of binding sites for 56 (JRC), 57 (UKK) and 66 (UKN1) TFs. Twenty-five TFBSs overlapped between all samples treated with VPA (Fig. 10a), that is, there was a relatively high overlap of responses on the level of TFBS. A similar behaviour was observed after treatment with MeHg: less than 10 % of the PS overlapped between UKN1 and UKK. Amongst these PS, 46 TFBS (UKN1) or 44 TFBS (UKK) were overrepresented and out of these, twenty (>40 %) overlapped (Fig. 10b).

In view of these findings, it was interesting to look at an overlap of transcriptome changes common to each of the toxicants in all test systems. We identified the PS and TFBS jointly modified in all three test systems by VPA or in UKN1 and UKK by MeHg. Only 3 (0.5 %) of the PS generally altered by VPA were also significantly affected by MeHg (Fig. 10c). In contrast, more than 50 % of all TFBS common to MeHg or VPA overlapped also between the two compounds (Fig. 10c). The large overlap of commonly enriched TFBS between all test systems and compounds provides evidence for the existence of a set of ‘common transcription factors’ (including, e.g., E2F, ETF, SP1 and AP-2 (Fig. S8). The only TFBS enriched by all VPA treatments, but not MeHg, was the homeobox gene Hmx3 (also known as NKX5.1). The only TFBS enriched by all MeHg treatments, but not VPA, was the one for GCM transcriptional regulators (Fig. S8).

Similar comparisons of compound responses were also performed in individual test systems; for instance, in UKK, only 205 PS of the 3,892 PS regulated by VPA overlapped with those affected by MeHg (Fig. 10d). On the level of TFBS, the overlap was much larger, as 22 of the 57 TFBS enriched in the genes regulated by VPA, were also found for MeHg (Fig. S9A).

Treatment of the UKN1 test system with VPA or MeHg resulted in the regulation of genes associated with 66 TFBS in their promoter in the case of VPA and 46 TFBS in the case of MeHg. Of these, 29 (comprising, e.g., AP-2, EGR, STAT1, HIF-1, AhR and Sp1) were similar for both compounds, 37 (comprising, e.g., HSF-1, IRF-1, PAX5 and NKX2-5) were specific for VPA, and 17 (comprising, e.g., ATF4, HOXA4 and ZIC2) specific for MeHg (Fig. S9B). Again, the overlap of TFBS was much larger than the one of individual PS. Only 142 of the 3,697 genes regulated by VPA overlapped with those affected by MeHg (Fig. 10e).

Besides the commonly regulated TFBS, we found for each compound also TFBS that were specific for the test system and the chemical used. These may be used as signatures for related chemicals within one class, while the commonly affected TFBS may give a general indication of toxicity (Supplementary Table S2). In conclusion, a remarkable observation of the present study is that the TFBS showed an astonishingly large overlap in view of the very small overlap on the level of the individual genes. Analysis of further compounds is required to determine whether the emerging concept of a ‘common toxic response TFBS’ and a ‘compound-specific TFBS’ is universal.

## Electronic supplementary material

Below is the link to the electronic supplementary material.

Supplementary material 1 (PDF 1104 kb)

Supplementary material 2 (XLS 2231 kb)

## Abbreviations

PS: Probe sets
DMA: DNA microarray
BMC: Benchmark concentration
TFBS: Transcription factor binding site
GO: Gene ontology
