# Supplementary material for: Human embryonic stem cell-derived test systems for developmental neurotoxicity: a transcriptomics approach
Source: Arch Toxicol. 2012 Nov 21;87(1):123–43. doi: 10.1007/s00204-012-0967-3 (PMC3535399; doi:10.1007/s00204-012-0967-3)
Supplement: Supplementary file 1 — Supplementary material 1 (PDF 1104 kb) [file 204_2012_967_MOESM1_ESM.pdf]

Supplementary figures

Overview:

Fig S1 **Determination of the test concentrations for DNA microarray analysis (DMA) – Page (P) . 1**

Fig S2 **Differential alterations of gene expression by valproic acid (VPA) and methyl mercury (MeHg). – P. 2**

Fig S3 **Volcano plot analysis – P. 3**

Fig S4 **Principal component analysis (PCA) of regulated genes in several test systems after subtraction of controls – P. 4-7**

Fig S5 **Simulation of different numbers of experiments (pairs of DMA) and their impact on the numbers of significantly-regulated probe sets (PS) – P. 8**

Fig S6 **Schematic representation of the PK model developed for VPA – P. 9-10**

Fig S7 **Overrepresented gene ontology groups – P. 11-12**

Fig S8 **Enrichment of transcription factor binding sites (TFBS) amongst toxicant-regulated genes – P.13**

Fig S9 **Comparison of MeHg and VPA responses in the UKN1 and UKK test system with respect to transcription factor binding site (TFBS) enrichment – P.14-15**

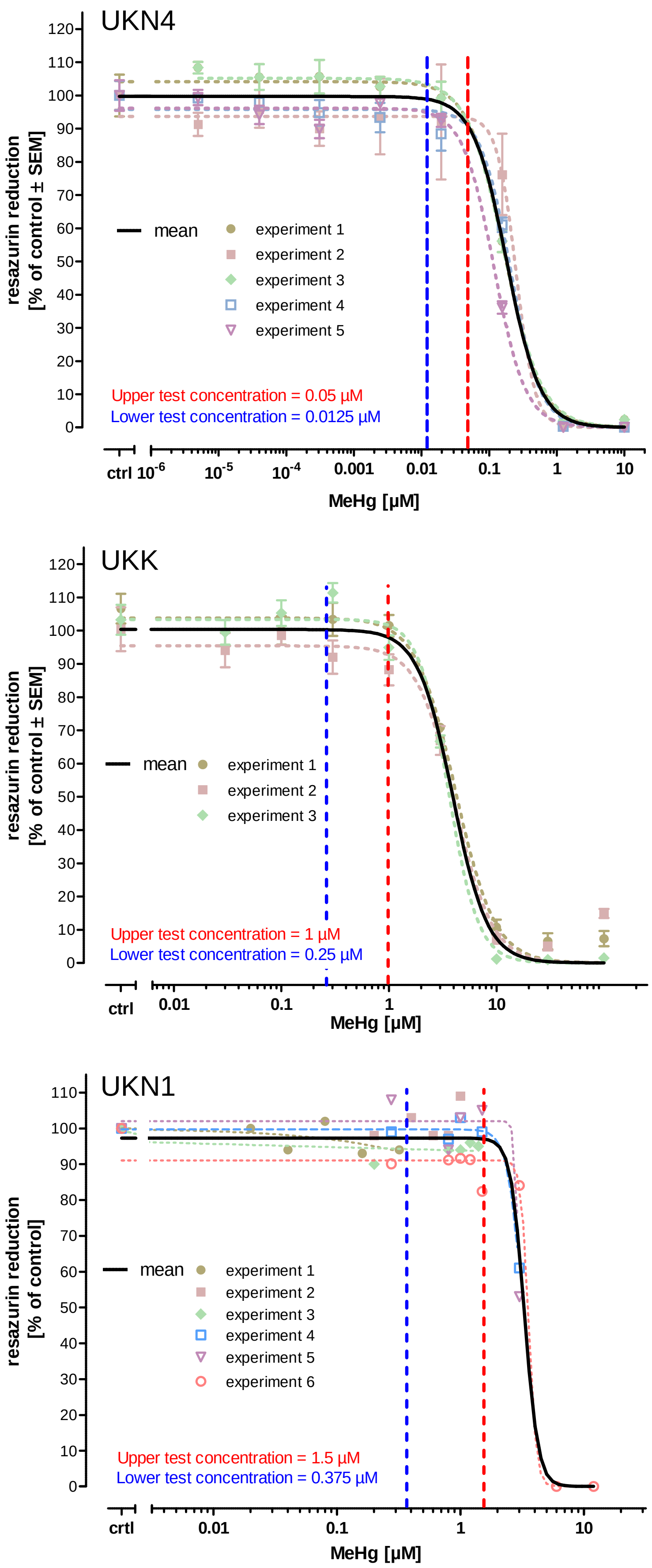

Fig. S1: Determination of the test concentrations for DNA microarray analysis.

Cells were treated with MeHg at the concentrations indicated, under conditions described for each test system in Fig. 1. At the end of the incubation period, cell viability was determined by the resazurin reduction assay. Data are normalized to untreated control cultures which were defined as 100%. The data points are averages ± SEM from three technical replicates. Each of the experiments was repeated several times (indicated by different color codings) with different cell preparations. The data from the different biological experiments were averaged (black line). To determine the "highest non-cytotoxic concentration", the BMC was determined graphically, taking the variation of individual experi-mental systems into account. This "upper test concentration" (= BMC) of the drug is indicated by the red dashed line. The "lower test concentration" (LOW) was determined by dividing the BMC by a factor of four. This is indicated by a blue dashed line.

Differential alterations of gene expression

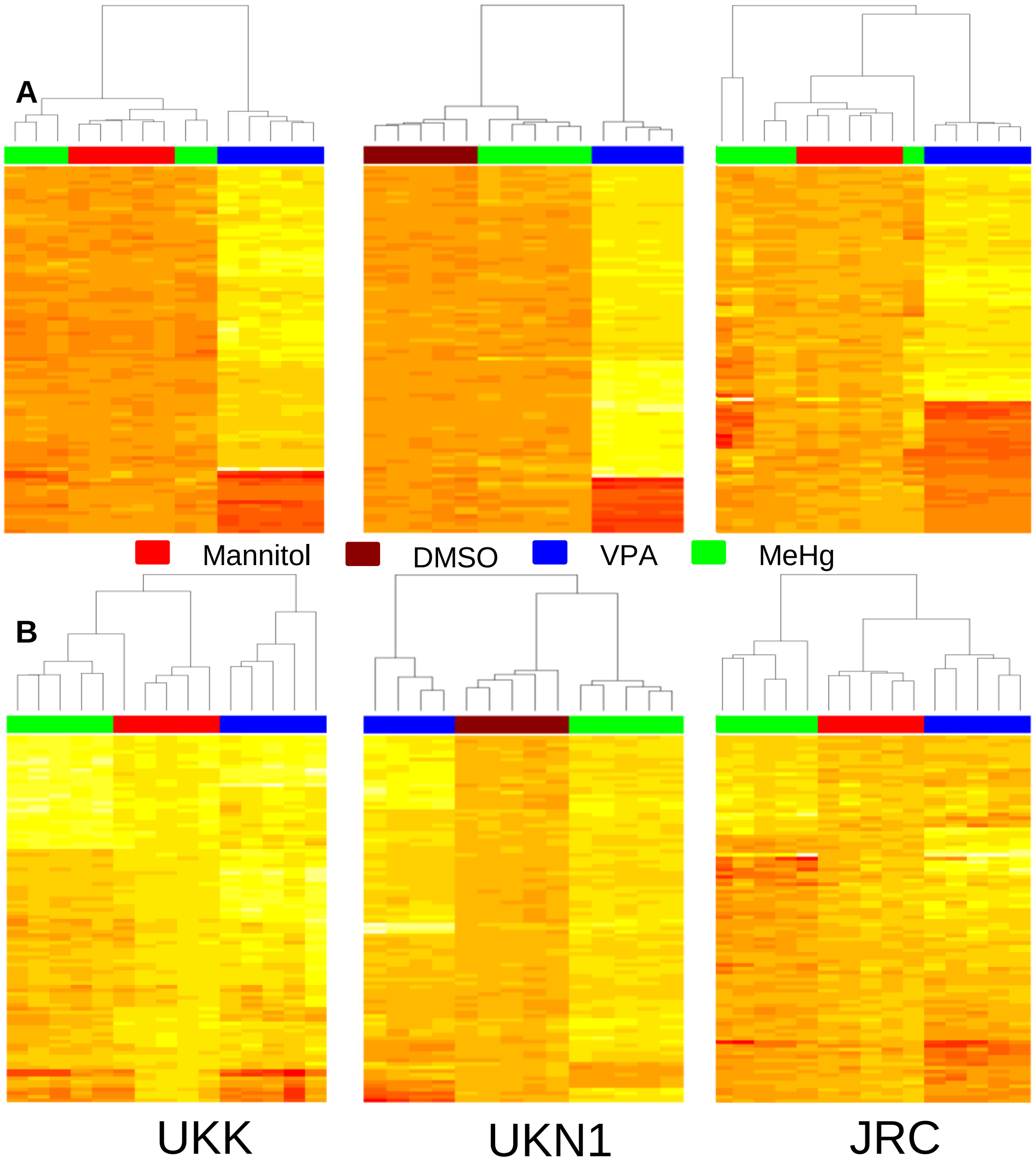

Fig. S2: Differential alterations of gene expression by valproic acid (VPA) and methyl mercury (MeHg).

Three different test systems (UKK, UKN1, JRC) were exposed to VPA (blue label on top of the heatmap) or MeHg (green label), at their respective bench mark concentration, or to D-mannitol (red). The differentially expressed genes (vs untreated controls) were determined in 4-5 independent experiments (shown as lanes of the heatmaps). The similarity of the gene expression patterns is indicated by the Pearson's distance dendrogram on top. The heatmaps are based on 100 selected genes.

A. These comprise the 100 genes with the lowest adjusted p-values according to the Limma t-Test for regulation by VPA. **B**. These comprise the 100 genes with the lowest adjusted p-values according to the Limma t-Test for regulation by MeHg.

The colors of the heatmap (yellow → red glow lookup table) indicate the relative gene regulation level above or below the average for each row.

Volcano plot analysis of gene array data

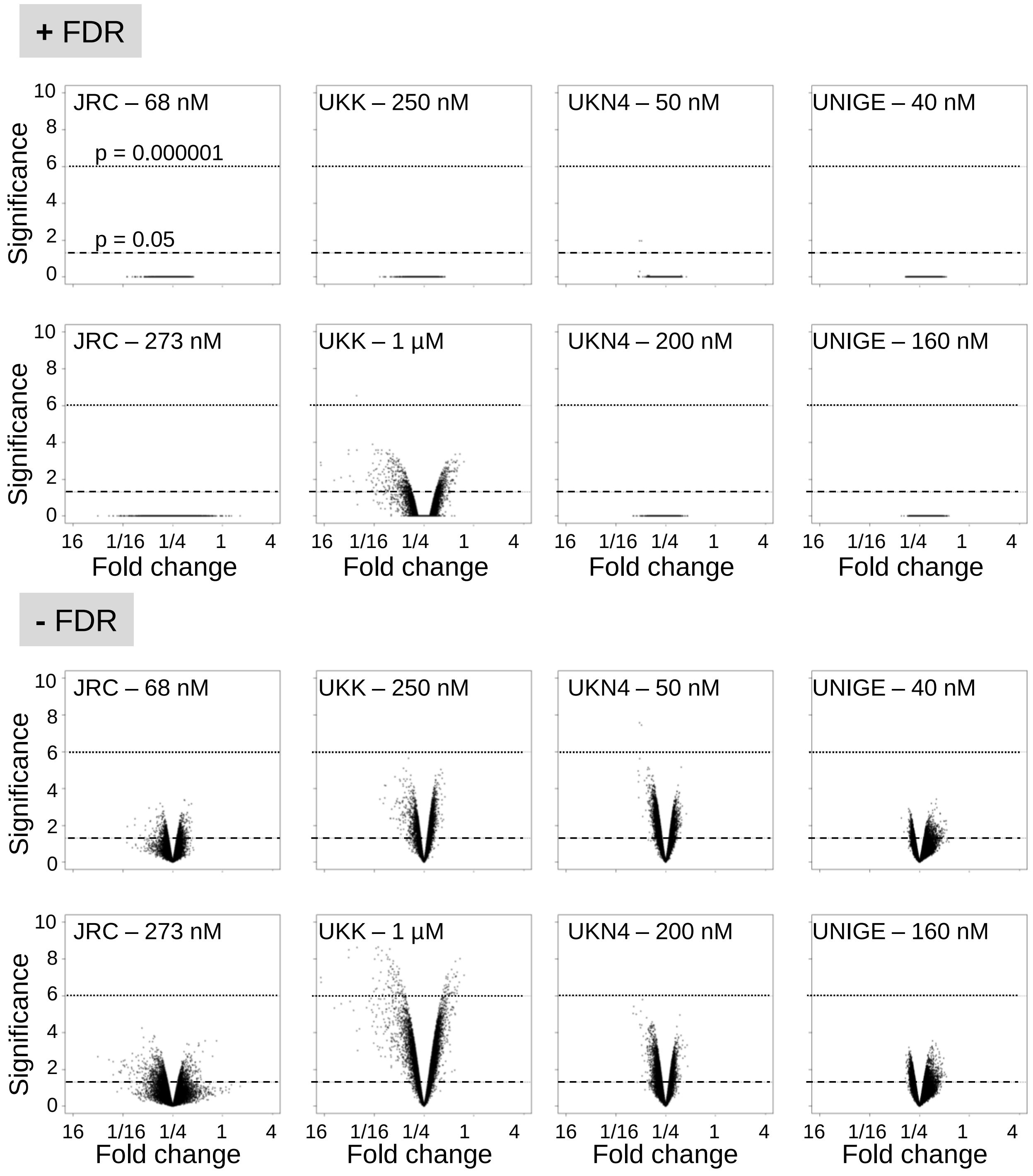

Fig. S3 Volcano plot analysis of gene array data after incubation of the test systems UKK, JRC, UNIGE-1 and UKN 4 with MeHg.

Data were generated and calculated for each combination of test system and compound, as illustrated in Fig. 3. In the volcano-plot diagrams, fold-changes of the compound-induced gene expression are shown on the x-axis (log2-scale). The y-axis shows negative logarithmic adjusted p-values of a LIMMA t-test ($-\log_{10}$(p-value)). The p-values were **A.** FDR adjusted, or **B.** not FDR adjusted. The dashed lines show the p = 0.05 significance level and the dotted lines the p = 0.000001 significance level for optical guidance.

Fig. S4 Principal component analysis (PCA) of regulated genes in several test systems after subtraction of controls

The signal of all PS was determined in five different test systems (UKK, UKN1, JRC, UKN4 and UNIGE) after incubation with compounds as in Fig. 3. Then, the values for the respective controls were subtracted from the values of the DMA treated with VPA at the BMC (large blue) or at the LOW concentration (small blue dots), or MeHg (large and small green dots), or D-mannitol (red) or DMSO (black). These data were then used for PCA analysis. The lower right panel shows all data together, the other panels show the data for individual test systems within the same axes as for all systems. Corresponding controls have been subtracted. The number of PS were now stepwise reduced retaining only the PS with highest variability. **A:** all probe sets (corresponds to Fig. 3C), **B:** 5000 probe sets, **C:** 1000 probe sets, **D:** 500 probe sets, **E:** 200 probe sets, **F:** 100 probe sets. Good separation results were still obtained using only 500 probe sets. Further reduction to e.g. 100 probe sets did no longer allow good separation.

Figures are displayed on pages 5-7

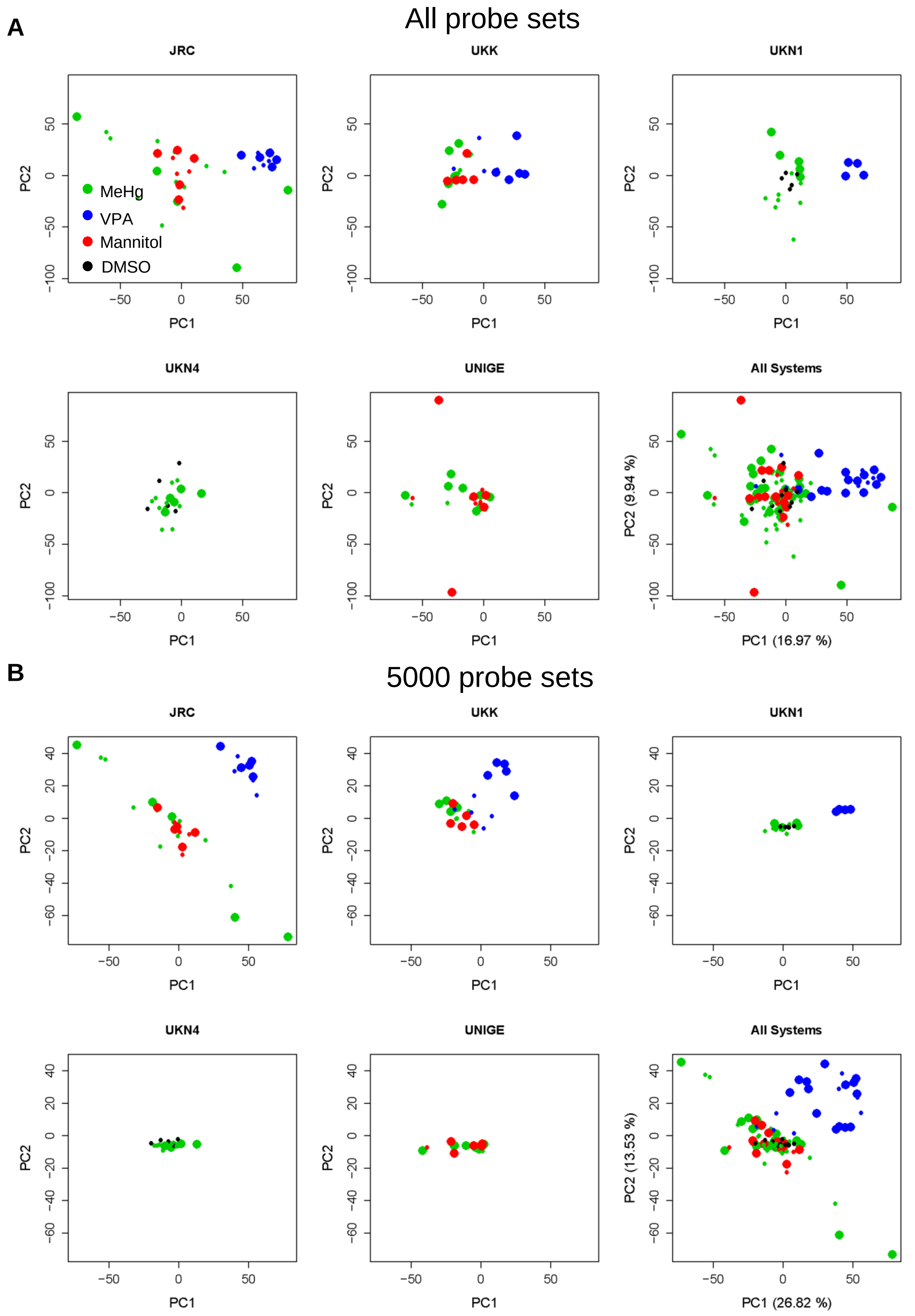
A
All probe sets
JRC
UKK
UKN1
UKN4
UNIGE
All Systems
MeHg
VPA
Mannitol
DMSO
PC1
PC2
PC1 (16.97 %)
PC2 (9.94 %)
B
5000 probe sets
PC1 (26.82 %)
PC2 (13.53 %)

Fig. S4

C 1000 probe sets

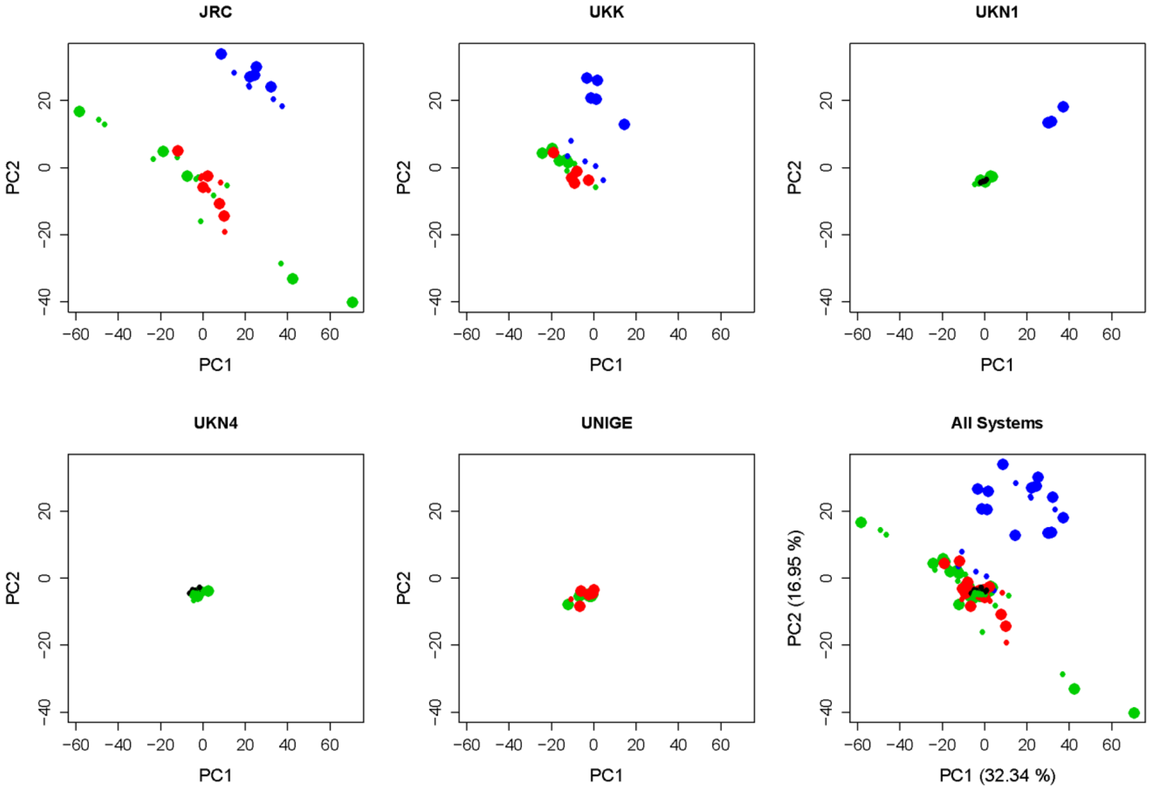

D 500 probe sets

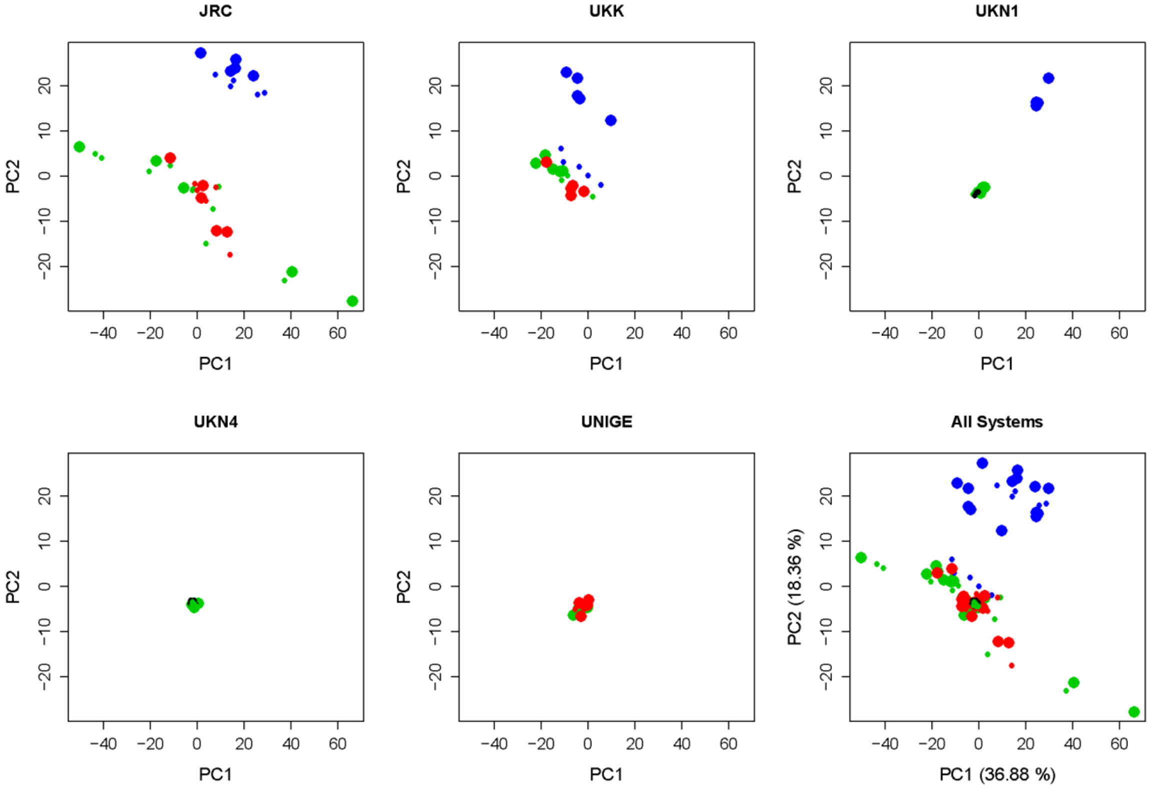

Fig. S4

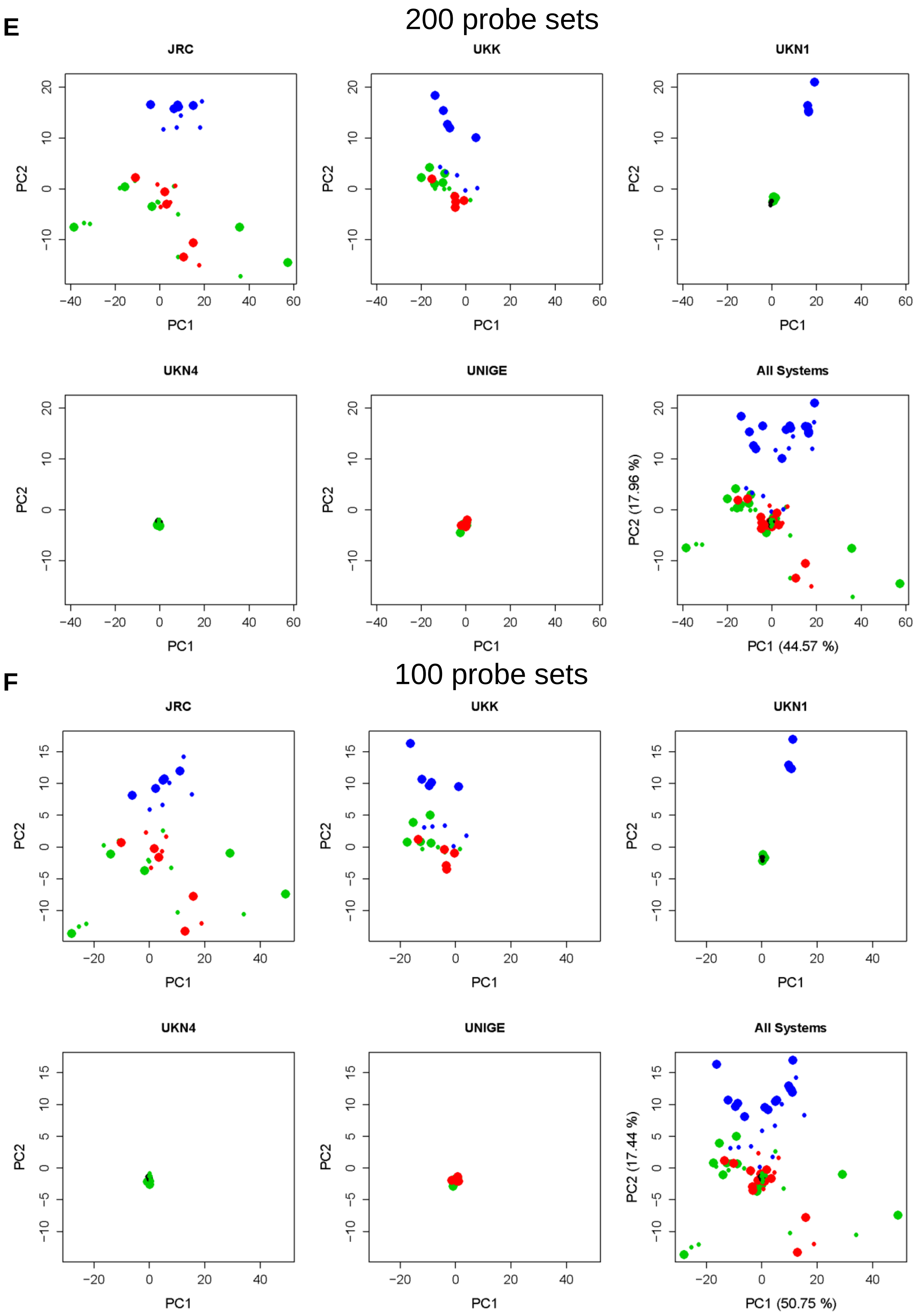
E
200 probe sets
JRC
UKK
UKN1
UKN4
UNIGE
All Systems
PC1
PC2
PC1 (44.57 %)
PC2 (17.96 %)
F
100 probe sets
PC1 (50.75 %)
PC2 (17.44 %)

Fig. S4

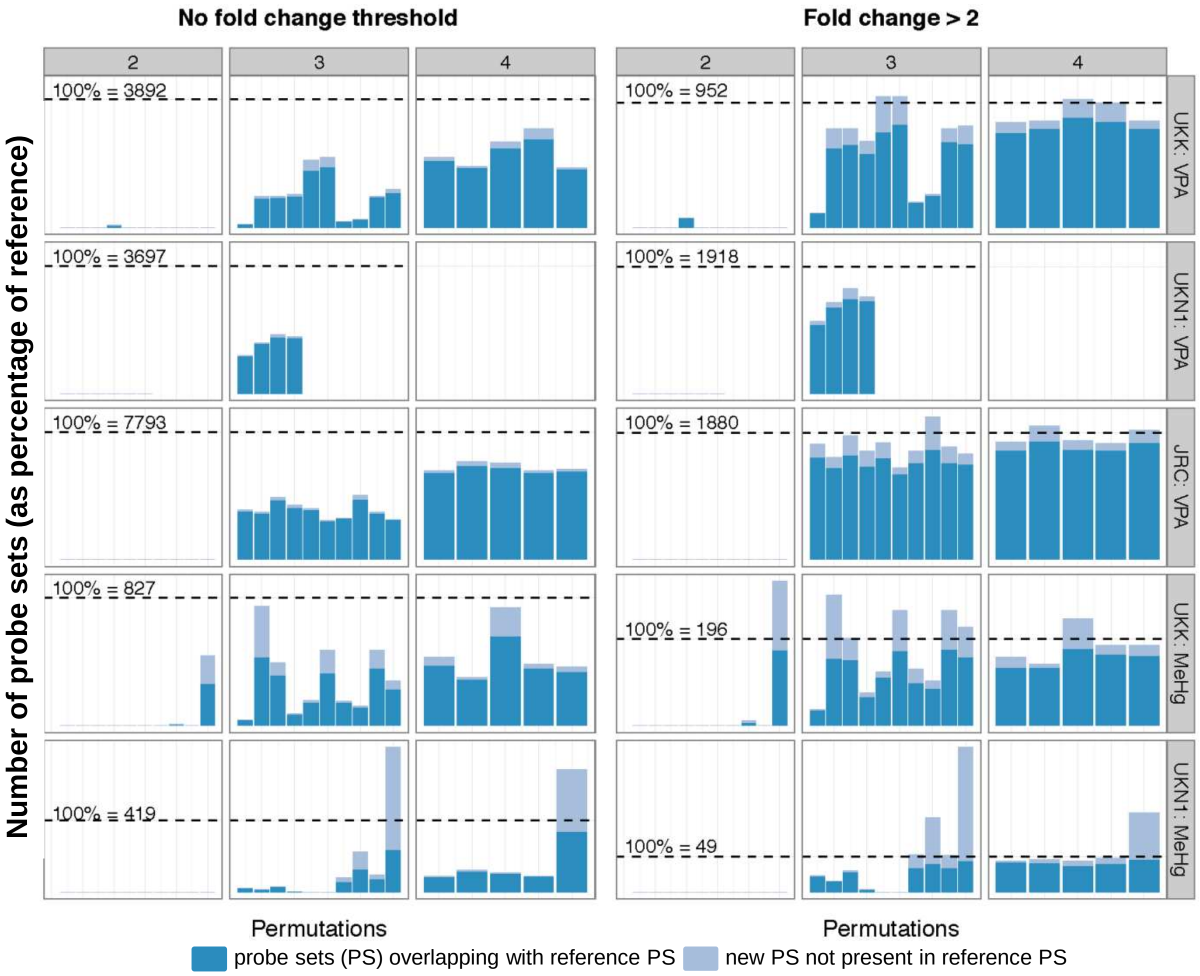

Fig. S5: Simulation of different numbers of experiments (pairs of DMA) and their impact on the numbers of significantly-regulated PS

VPA was tested in the test systems JRC und UKK at its BMC in 5 independent experiments, and in UKN1 in 4 experiments. MeHg was tested in UKN1 in 5 experiments. The number of significantly regulated genes (Benjamini-Yekutieli FDR correction) was calculated without further restrictions (left) or with the restrictions that PS should be regulated more than 2-fold (right). The numbers of PS are indicated above the dashed black lines and they were set as 100% reference points. The blue bars indicate how many of these PS were identified when different permutations of 2, 3 or 4 experiments (indicated as grey headings) were used. The light blue bars indicate how many additional PS were identified, when only subsets of the original 5 (4) experiments were analyzed. For instance, the 5 bars in the panel with the coordinates 4/JRC:VPA represent the five possible ways of leaving out one of the experiments. The 10 bars in the panel with the coordinates 3/JRC:VPA represent the 10 possible permutations of leaving out 2 of the experiments and then recalculating the significant PS on the basis of the remaining 3 DMA.

Fig. S6A: Schematic representation of the PK model developed for VPA.

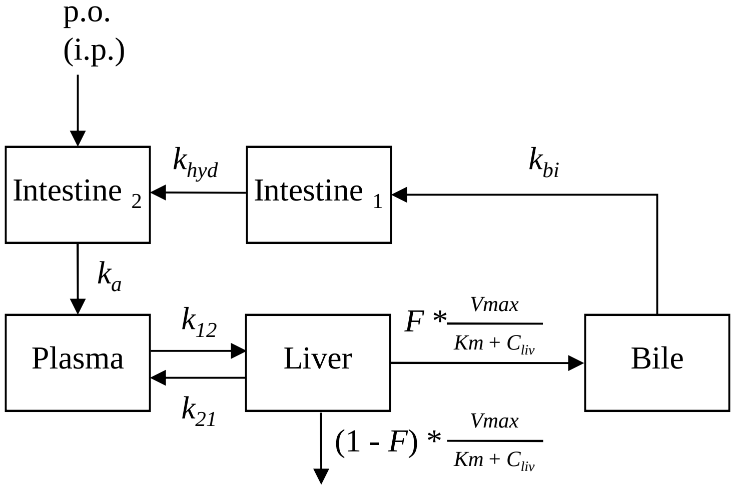

The following differential equations describe the rates of change of VPA (µg/min) in the model compartments, where A denote amounts in µg and C denote concentrations in µg/mL:

Plasma compartment

$$\frac{dA_{pl}}{dt} = k_a \times A_{in2} - k_{12} \times A_{pl} + k_{21} \times A_{liv}$$

$$C_{pl} = \frac{A_{pl}}{V}$$

Bile compartment

$$\frac{dA_{liv}}{dt} = k_{12} \times A_{pl} - k_{21} \times A_{liv} - \frac{V_{max}}{K_m + C_{liv}} \times A_{liv}$$

$$C_{liv} = \frac{A_{liv}}{V_{liv}}$$

Intestinal compartments

$$\frac{dA_{bi}}{dt} = F \times \frac{V_{max}}{K_m + C_{liv}} \times A_{liv} - k_{bi} \times A_{bi}$$

Liver compartment

$$\frac{dA_{in1}}{dt} = k_{bi} \times A_{bi} - k_{hyd} \times A_{in1}$$

$$\frac{dA_{in2}}{dt} = k_{hyd} \times A_{in1} - k_a \times A_{in2}$$

Values for the following parameters were obtained by fitting to data presented by Binkerd et al. (1988) and Kobayashi (1991):

k_a – absorption rate constant; 0.05 min^{-1}

k_{12} – plasma-to-liver transfer rate constant; 0.274 min^{-1}

k_{21} – liver-to-plasma transfer rate constant; 0.279 min^{-1}

V – (initial) volume of distribution; 51.6 mL

V_{liv} – liver compartment volume; 12.3 mL

V_{max} – maximum velocity bile excretion; 25.2 µg/mL/min

K_m – Michaelis constant bile excretion; 362 µg/mL

F – fraction excreted into bile; 0.18

k_{bi} – rate constant for bile flow to intestine; 0.0033 min^{-1}

k_{hyd} – rate constant for hydrolysis of glucuronidated VPA; 0.0062 min^{-1}

Fig. S6B: Estimated lipid content and albumin concentration in *in vitro* test media

Medium	lipid content	albumin concentration
	[mg/l]	*[µM]*
**UKK**	120	244.3
**UKN1**	92	184.7
**JRC**	2.8	5.7
**UNIGE***	2.9	5.8
**UKN4**	2.9	5.8
**Rat plasma**	3600	421.0
**Human plasma**	6000	600

Fig. S6B: Estimated lipid content and albumin concentration in *in vitro* test media as well as rat and human plasma

Test medium lipid content and albumin concentrations were calculated on the basis of available supplier information on medium constituents. The data on rat plasma used in the present *in vitro-in vivo* correlation have been adopted from Verwei et al. (2006). The original references are Barber et al. (1990) for albumin, and DeJongh et al (1997) for lipids. Human plasma values are mentioned for comparison and were taken from Gülden and Seibert (2003). The original data on albumin are from Lindup et al (1987) and for lipids from Patterson et al (1988). Note that plasma lipid content is highly dependent on diet. Rat values are assumed to reflect average values on standard chow, human values are average values under fasting conditions.

* B27 medium composition is not disclosed; by assumption the same albumin and lipid concentrations as DMEM/F12 were used.

Barber, B.J., Schultz, T.J., Randlett, D.L., 1990. Comparative analysis of protein content in rat mesenteric tissue, peritoneal fluid and plasma. Am. J. Physiol. Gastrointest. Liver Physiol. 258, G714–G718.

DeJongh, J., Verhaar, H.J.M., Hermens, J.L.M., 1997. A quantitative property–property r elationship (QPPR) approach to estimate in vitro tissue-blood partition coefficients or organic chemicals in rats and humans. Arch. Toxicol. 72, 17–25.

Gulden M, Seibert H (2003) In vitro-in vivo extrapolation: estimation of human serum concentrations of chemicals equivalent to cytotoxic concentrations in vitro. Toxicology 189(3):211-22

Lindup, W.E., 1987. Plasma protein binding of drugs: some basic and clinical aspects. In: Bridges, J.W., Chasseaud, L.F., Gibson, G.G. (Eds.), Progress in Drug Metabolism, vol. 10. Taylor and Francis, London, pp. 141-185.

Patterson, D.G., Jr., Needham, L.L., Pirkle, J.L., Roberts, D.W., Bagby, J., Garrett, W.A., Andrews, J.S., Falk, H., Bernert, J.T., Sampson, E.J., Houk, V.N., 1988. Correlation between serum and adipose tissue levels of 2,3,7,8-tetrachlorodibenzo-p -dioxin in 50 persons from Missouri. Arch. Environ. Contam. Toxicol. 17, 139-143.

Verwei M, van Burgsteden JA, Krul CA, van de Sandt JJ, Freidig AP (2006) Prediction of in vivo embryotoxic effect levels with a combination of in vitro studies and PBPK modelling. Toxicol Lett 165(1):79-87

Fig. S7 Overrepresented gene ontology groups

A g:Profiler query (Reimand et al. 2007) was initially made, and only results from the biological process and pathway branches were retained. These were viewed as a subgraph of the whole gene ontology tree. All categories were deleted that were larger than 1000 genes and smaller than 50 genes. Then, connected components from the remaining graph were identified, and from each of these, the category with the highest p-value was selected. These were ordered by p-value and the top 40 are displayed. When displaying the categories, the font sizes were first scaled to be proportional to the log10 of enrichment p-value. To enable global comparison, the grey shade of the letters was scaled the same way over all plotting windows.

The figure is displayed on page 12

Reimand J, Kull M, Peterson H, Hansen J, Vilo J (2007) g:Profiler--a web-based toolset for functional profiling of gene lists from large-scale experiments. Nucleic Acids Res 35(Web Server issue):W193-200

Fig. S7: Overrepresented gene ontology groups

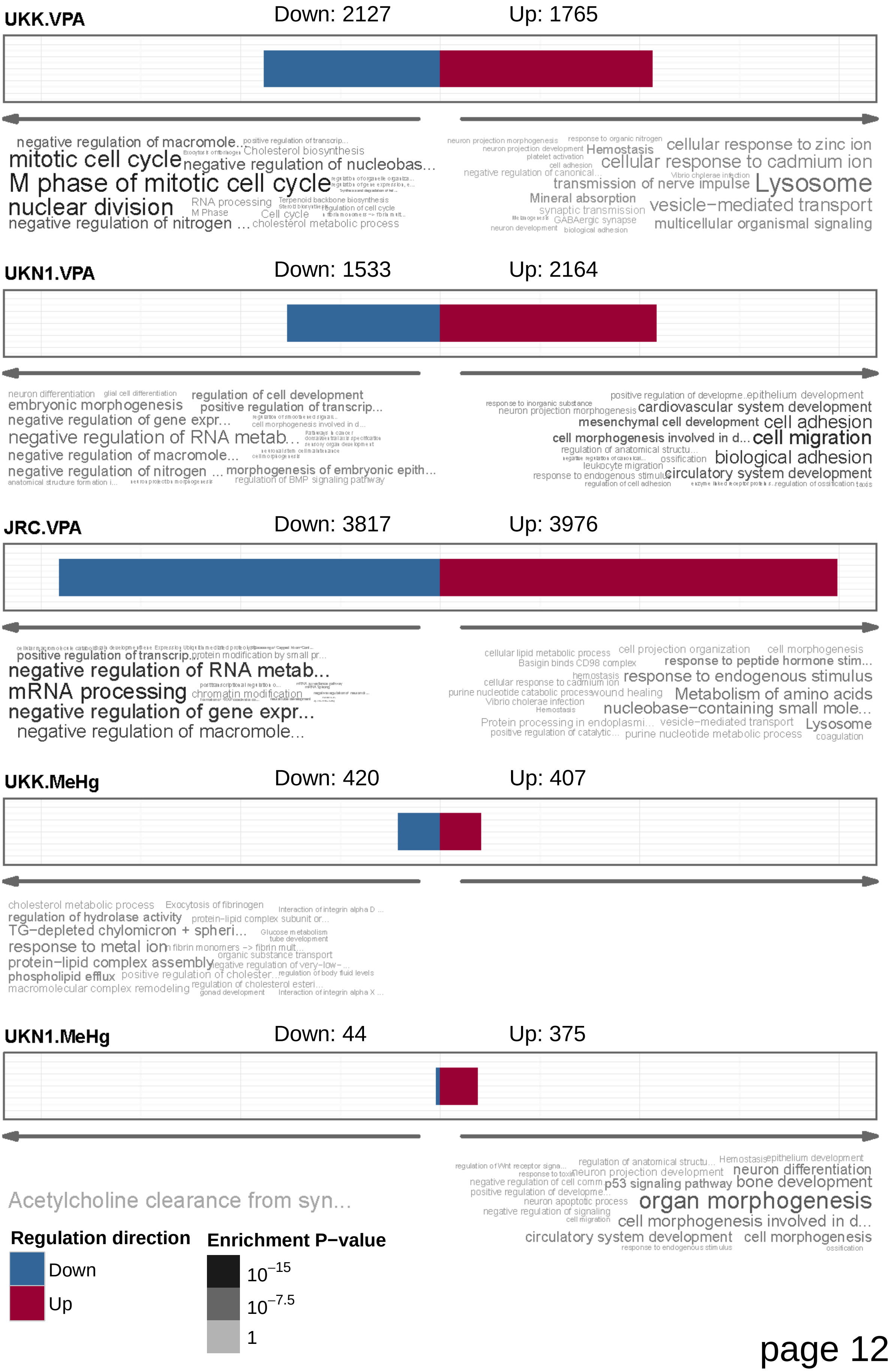

Fig. S8: Enrichment of transcription factor binding sites

TFBS	VPA UKN1	VPA UKK	VPA JRC	MeHg UKN1	MeHg UKK
MOVO-B	■	■	■	■	■
SRY	■	■	■	■	■
Sp1	■	■	■	■	■
E2F	■	■	■	■	■
MAZ	■	■	■	■	■
EGR	■	■	■	■	■
ZF5	■	■	■	■	■
HIC1	■	■	■	■	■
UF1H3[b]	■	■	■	■	■
c-Myc:Max	■	■	■	■	■
ZNF219	■	■	■	■	■
HIF-1	■	■	■	■	■
E2F-1	■	■	■	■	■
AP-2	■	■	■	■	■
ETF	■	■	■	■	■
AhR:Amt	■	■	■	■	■
MTF-1	■	■	■	□	■
FOXP1	■	■	■	□	■
Egr-1	■	■	■	■	□
MZF1	■	■	■	■	□
Pax-4	■	■	■	■	□
STAT1	■	■	■	■	□
AP-2[a]	■	■	■	■	□
AHRHIF	■	■	■	■	□
Hmx3	■	■	■	□	□
VDR	■	□	□	■	■
GCM	□	□	□	■	■
Nkx6-2	□	■	□	■	■
Oct-1	□	■	□	■	■

■ enriched □ not enriched

Fig. S8: Enrichment of transcription factor binding sites (TFBS) amongst toxicant-regulated genes. The test systems UKN1, JRC and UKK were exposed to VPA and UKN1 and UKK were also exposed to MeHg. Both toxicants were applied at their BMCs and the significantly regulated genes were identified as in Fig. 3. The overrepresented TFBS in these sets of genes were determined with the PRIMA algorithm (see methods). Black-filled cells in the table indicate treatments (column titles) in which the TFBS (row titles) was enriched (PRIMA $p<0.05$).

MeHg VPA

TFBS (MeHg only)	
TF ID*	TF full Name**
AREB6	ZEB1: zinc finger E-box binding homeobox 1 (HGNC)
c-Myb	v-myb myeloblastosis viral oncogene homolog (avian)
FOXJ2	Forkhead box J2
Freac-3	FOXC1: forkhead box C1(HGNC)
GCM	Glial cells missing factor A
Helios_A	IKZF2: IKAROS family zinc finger 2 (Helios) (HGNC)
HFH-1	FOXM1: forkhead box M1 (HGNC)
HNF-1	Hepatocyte nuclear factor (HNF) 1 homeobox A
HSF1	Heat shock transcription factor 1
IRF1	Interferon regulatory factor 1
KAISO	ZBTB33: zinc finger and BTB domain containing 33 (HGNC)
Lyf-1	Lymphoid transcription factor 1
Pax-5	Paired box 5
Pit-1	Pituitary-specific factor 1
PLZF	Promyelocytic leukemia zinc finger
STAT	Signal Transducer and Activator of Transcription
STAT4	Signal Transducer and Activator of Transcription 4
STATx	Family: signal transducers and activators of transcription
SZF1-1	ZNF589: zinc finger protein 589 (HGNC)
TBX5	T-box protein 5
VDR	Vitamin D receptor
VDR,_CAR,_PXR	VDR: vitamin D receptor; CAR: constitutive androstane receptor; PXR: pregnane X receptor

TFBS in both treatments	
TF ID*	TF full Name**
Oct-1	Octamer binding factor 1; also known as POU2F1: POU class 2 homeobox 1 (HGNC)
AhR:Arnt	Aryl hydrocarbon receptor: Aryl hydrocarbon receptor nuclear translocator
AIRE	Autoimmune regulator
AP-2	Activating protein 2
c-Myc:Max	v-myc myelocytomatosis viral oncogene homolog (avian) (HGNC): myc-associated factor X
E2F	E2F transcription factor
E2F-1	E2F transcription factor 1
EGR	Early Growth Responsive factor
ETF	EGFR-specific transcription factor
FAC1	Now known as BPTF: bromodomain PHD finger transcription factor (HGNC)
FOXP1	Forkhead box P1
HIC1	Hypermethylated in cancer 1
HIF-1	Hypoxia Induced Factor 1
MAZ	Myc-associated zinc finger protein
MOVO-B	Mouse homologue of Drosophila Ovo protein
MTF-1	Metal-regulatory transcription factor 1
Nkx6-2	NK6 homeobox 2
Sp1	Specificity protein 1
SRY	Sex-determining region Y
UF1H3BETA	Uf1h3beta transcription factor
ZF5	Zinc finger protein 5
ZNF219	Zinc finger protein 219

TFBS (VPA only)	
TF ID*	TF full Name**
AFP1	Alpha fetoprotein enhancer binding protein
AhR	Aryl hydrocarbon receptor
AHRHIF	Aryl hydrocarbon receptor, hypoxia inducible factor
AP-2alpha	Activating protein 2 alpha
AP-2alphaA	Activating protein 2 alphaA
ATF	Activating transcription factor
ATF4	Activating transcription factor 4 (tax-responsive enhancer element B67)
Brn-2	Brain-2; known as POU3F2: POU class 3 homeobox 2 (HGNC)
CBF	Core binding factor
CDP_CR1	Cut-like homeodomain protein
Egr-1	Early Growth Responsive factor 1
GZF1	GDNF-inducible zinc finger protein 1 (HGNC)
Hmx3	H6 family homeobox 3
HOXA3	Homeobox A3
Ik-1	Ikaros 1 transcription factor
Ik-3	Ikaros 3 transcription factor
IPF1	Insulin promoter factor 1
IRF-1	Interferon regulatory factor 1
MEF-2	Myocyte Enhancer Factor 2
MZF1	Myeloid zinc finger 1
Nanog	Nanog homeobox
NF-Y	Nuclear factor Y (Y-box binding factor)
NKX3A	Now known as NKX3-1: NK3 homeobox 1 (HGNC)
Nrf-1	Nuclear respiratory factor 1
Pax-1	Paired box gene 1
Pax-3	Paired box gene 3
Pax-4	Paired box gene 4
Pax-6	Paired box gene 6
S8	S8 homeobox
STAT1	Signal Transducer and Activator of Transcription 1
STAT5A	Signal Transducer and Activator of Transcription 5A
Tax/CREB	Tax: now known as CNTN2: contactin 2 (axonal) (HGNC)/ CREB: cyclic AMP response element-binding protein
Tst-1	Now known as POU3F1: POU class 3 homeobox 1 (HGNC)
USF	Upstream stimulatory factor
Whn	Winged-helix nude

*Transcription factor (TF) ID provided by PRIMA/Expander

**source was usually TRANSFAC Public, release 7.0. When appropriate, updated names from the HUGO Gene Nomenclature Committee, www.genenames.org, are provided, indicated by (HGNC)

Fig. S9A: Comparison of MeHg and VPA responses with respect to transcription factor binding site (TFBS) enrichment

The test system UKK was exposed to MeHg (1 µM) or VPA (2 mM), and the significantly regulated probe sets were determined (as reported in Fig. 3). Statistical overrepresentation of TF-binding sites (TFBS) in the promoters of the regulated genes was determined with the PRIMA algorithm for both treatments. TFBS were grouped into those only found enriched for MeHg treatment (red box), those only found for VPA treatment (blue box) and those found to be enriched by both compounds (purple box).

MeHg VPA

TFBS (MeHg only)

TF ID*	TF full Name**
ATF	Activating transcription factor
ATF4	Activating transcription factor 4
C/EBPdelta	CCAAT-enhancer-binding protein delta
GCM	Glial cells missing factor A
HNF4	Hepatocyte nuclear factor 4
HOXA4	Homeobox A4
LEF1	Lymphoid enhancer-binding factor 1
LRF	Leukemia/lymphoma-related factor
MAF	v-maf musculoaponeurotic fibrosarcoma oncogene homolog (avian) (HGNC)
Nkx6-2	NK6 homeobox 2
N-Myc	v-myc related oncogene, neuroblastoma derived
Oct-1	Octamer binding factor 1; also known as POU2F1: POU class 2 homeobox 1 (HGNC)
Sp3	Stimulating Protein 3
SRF	Serum response factor
Tax/CREB	CNTN2: contactin 2/CREB: cyclic AMP response element-binding protein
TFIIA	General transcription factor IIA
Zic2	Zinc finger protein of the cerebellum 2

TFBS in both treatments

TF ID*	TF full Name**
AhR:Arnt	Aryl hydrocarbon receptor: Aryl hydrocarbon receptor nuclear translocator
AHR:HIF	Aryl hydrocarbon receptor, hypoxia inducible factor
AP-2	Activating protein 2
AP-2alpha	Activating protein 2 alpha
CAC-BP	CAC-binding protein
c-Myc:Max	v-myc viral oncogene homolog: myc-associated factor X
E2F	E2F transcription factor
E2F-1	E2F transcription factor 1
EGR	Early Growth Responsive factor
Egr-1	Early Growth Responsive factor 1
ETF	EGFR-specific transcription factor
HIC1	Hypermethylated in cancer 1
HIF-1	Hypoxia Induced Factor 1
MAZ	Myc-associated zinc finger pr.
MAZR	MAZ related factor
MEF-2	Myocyte Enhancer Factor 2
MOVO-B	Homologue of Ovo protein
MZF1	Myeloid zinc finger 1
P300	E1A-associated protein p300
Pax-4	Paired box gene 4
RREB-1	Ras-responsive element binding protein 1
Sp1	Specificity protein 1
SRY	Sex-determining region Y
STAT1	Signal transducer and activator of transcription 1
TFII-I	General Transcription Factor II-I
UF1H3BETA	Uf1h3beta transcription factor
VDR	Vitamin D receptor
ZF5	Zinc finger protein 5
ZNF219	Zinc finger protein 219

TFBS (VPA only)

TF ID*	TF full Name**
Alx-4	Aristaless homeobox like 4
aMEF-2	myocyte-specific enhancer factor, alternatively spliced exon
BLIMP1	B lymphocyte induced maturation protein 1; now known as PRDM1
CDP	CCAAT displacement protein
CDX	Caudal type homeobox
CHOP:C/EBPalpha	CAAT/enhancer binding protein homologous transcription factor: CCAAT Enhancer Binding Protein alpha
CHX10	Now known as ZSX2: visual system homeobox 2 (HGNC)
CP2	Now known as TFCP2:; transcription factor CP2 (HGNC)
DBP	DNA binding protein
ELF-1	Enhancer Lymphocyte Factor 1
FAC1	Now known as BPTF: bromodomain PHD finger transcription factor (HGNC)
FOXO4	Forkhead box O4 (HGNC)
FOXP1	Forkhead box P1
Freac-3	known as FOXC1: forkhead box C1
HEN1	Now known as NHLH1: nescient helix loop helix 1
HFH-1	Now known as FOXM1: forkhead box M1
Hmx3	H6 family homeobox 3
HSF1	Heat shock transcription factor 1
Ik-3	Ikaros 3 transcription factor
IRF-1	Interferon regulatory factor 1
IRF-7	Interferon regulatory factor 7
ISRE	Interferon-stimulated response element
Lyf-1	Lymphoid transcription factor 1
MTF-1	Metal-regulatory transcription factor 1
myogenin_/_NF-1	MyoG; myogenin (myogeninc factor 4) (HGNC) _/_ nuclear factor 1
NF-kB (p50)	Nuclear Factor kappa B, p50
Nkx2-2	NK2 homeobox 2
Nkx2-5	NK2 homeobox 5
NkX6-1	NK6 homeobox 1
NRSF	Neuron-restrictive silencer factor
Olf-1	ZNF423; zinc finger protein 423 (HGNC)
OTX	Orthodenticle related homeobox protein 1
Pax-5	Paired box 5
PU.1	PUrine-box binding factor 1
Sox-5	SRY (sex determining region Y)-box 5
TEF-1	Transcriptional enhancer factor 1
TTF-1	Thyroid transcription factor 1

*Transcription factor (TF) ID provided by PRIMA/Expander

**source was usually TRANSFAC Public, release 7.0. When appropriate, updated names from the HUGO Gene Nomenclature Committee, www.genenames.org, are provided, indicated by (HGNC)

Fig. S9B: Comparison of MeHg and VPA responses with respect to transcription factor binding site (TFBS) enrichment

The test system UKN1 was exposed to MeHg (1.5 µM) or VPA (0.6 mM), and the significantly regulated probe sets were determined (as reported in Fig. 3). Statistical overrepresentation of TF-binding sites (TFBS) in the promoters of the regulated genes was determined with the PRIMA algorithm for both treatments. TFBS were grouped into those only found enriched for MeHg treatment (red box), those only found for VPA treatment (blue box) and those found to be enriched by both compounds (purple box).
